# Characterizing Emerging Detector Materials for Low‐Dose X‐Ray Imaging

**DOI:** 10.1002/adma.202512795

**Published:** 2025-09-11

**Authors:** Kostiantyn Sakhatskyi, Vitalii Bartosh, Ying Zhou, Gebhard J. Matt, Jingjing Zhao, Sergii Yakunin, Jinsong Huang, Maksym V. Kovalenko

**Affiliations:** ^1^ Laboratory of Inorganic Chemistry Department of Chemistry and Applied Biosciences ETH Zürich Zürich CH‐8093 Switzerland; ^2^ Laboratory for Thin Films and Photovoltaics Empa – Swiss Federal Laboratories for Materials Science and Technology Dübendorf CH‐8600 Switzerland; ^3^ Department of Applied Physical Sciences University of North Carolina at Chapel Hill Chapel Hill NC 27599 USA; ^4^ School of Physical Science and Technology Chongqing Key Lab of Micro&Nano Structure Optoelectronics Southwest University Chongqing 400715 China; ^5^ Department of Chemistry University of North Carolina at Chapel Hill Chapel Hill NC 27599 USA; ^6^ Institute of Energy Science and Technology (SIEST) Sungkyunkwan University (SKKU) 2066, Seobu‐ro, Jangan‐gu Suwon Gyeonggi‐do 16419 Republic of Korea

**Keywords:** detective quantum efficiency, lead halide perovskites, low‐dose imaging, medical imaging, metal halide scintillators, noise equivalent dose, X‐ray detectors

## Abstract

Modern clinical diagnostics significantly rely on X‐ray medical imaging detectors, which play a key role in obtaining high‐quality images while ensuring patient radiation exposure adheres to the “as low as reasonably achievable” principle. The last decade has seen a renewed exploration of promising materials for X‐ray detection, foremost focusing on lead‐based perovskites and other metal halides as direct‐conversion semiconductors and scintillators. However, the reported performance characteristics, particularly X‐ray sensitivity and the limit of dose rate detection, are often incomplete or misleading for assessing the practical utility of materials. This perspective surveys various approaches to the X‐ray detector characterization of emerging materials, specifically focusing on Detective Quantum Efficiency within the context of low‐dose medical imaging applications. Guidelines are provided for choosing, estimating, and presenting the relevant figures of merit, encompassing Detection Efficiency, Noise Equivalent Dose, response time, and spatial resolution, accompanied by ready‐to‐use computational tools, including a MATLAB application, a Mathcad worksheet, and an interactive website.

## Graphical abstract

X‐ray medical imaging requires detectors that deliver images at as low as reasonably possible dose. This perspective outlines a universal characterization framework of X‐ray imaging detectors, suitable for both emerging semiconductor and scintillator materials, introduces a novel unified figure of merit that links dose with resolution, and offers an open‐access calculator, enabling fair benchmarking of detector prototypes at early technology‐readiness levels.

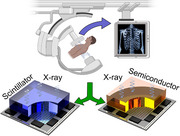

## 1 Introduction

Recent discoveries of novel direct‐conversion semiconductors^[^ ^1^, ^2^, ^3^, ^4^, ^5^ ^]^ and scintillators^[^ ^6^, ^7^, ^8^ ^]^ promise next‐generation X‐ray detectors capable of high‐quality imaging at significantly reduced radiation doses (*D*), offering substantial benefits for medical diagnostics. However, industrial and clinical adaptation of these emerging materials is hindered by a lack of consensus within the broad materials research community about which performance parameters and methods are most appropriate for detector characterization. Current standard industrial protocols,^[^ ^9^ ^]^ including IEC 62220‐1‐3,^[^ ^10^ ^]^ ASTM E2597/E2597M,^[^ ^11^ ^]^ and report of AAPM Task Group 116,^[^ ^12^ ^]^ primarily focus on benchmarking complete imaging systems at their final (above level 6) Technology Readiness Levels (TRLs).^[^ ^13^ ^]^ These standards are impractical for basic materials research (TRLs 1–2) and challenging to implement in early‐stage detector prototypes (TRLs 3–5). Consequently, the majority of the materials science community avoids these protocols, opting instead for simpler metrics such as X‐ray sensitivity^[^ ^14^ ^]^ and detection limit of dose rate (LoD),^[^ ^15^ ^]^ that often contradict the overarching goal of advancement in X‐ray medical detectors ‐ high‐quality imaging at low radiation doses.

In this Perspective, we survey characterization methods for X‐ray detectors, specifically focusing on the industry‐accepted Detective Quantum Efficiency (DQE) metric.^[^ ^9^, ^16^, ^17^, ^18^, ^19^ ^]^ We critically evaluate the benchmarking approaches commonly employed in materials research, analyze the key parameters influencing DQE, and propose guidelines for accurately assessing DQE in early‐stage detector prototypes. This framework encompasses materials used for both indirect and direct X‐ray sensing and is suitable for single‐channel detectors as well as multipixel imaging arrays. Furthermore, we propose a convenient new metric for benchmarking X‐ray detectors, integrating the radiation dose and spatial frequency dependencies of DQE. The calculation routine described in this Perspective is additionally provided as a Mathcad worksheet, a MATLAB application (see Data S1, Supporting Information), and as an interactive webpage.^[^ ^20^ ^]^ We also outline the fair approach with respect to the declared claims in the research articles, particularly in relation to different materials' development stages (**Table** **1**). The principal aim of this Perspective is to bridge the existing gap between established industrial‐standard methods and early‐stage development approaches by presenting a comprehensive characterization framework for low‐dose high‐quality X‐ray imaging, analogous to the frameworks successfully established in other material research fields, such as solar cells,^[^ ^21^ ^]^ thermoelectrics,^[^ ^22^ ^]^ photodetectors,^[^ ^23^ ^]^ solid‐state lighting,^[^ ^24^ ^]^ displays,^[^ ^25^ ^]^ batteries^[^ ^26^ ^]^ and lasers.^[^ ^27^ ^]^

**Table 1:** Recommendation for reporting material and detector metrics in a research article. The narrative and claims of the research article must be adjusted according to their TRL^[^ ^13a,b^ ^]^ so as not to misguide the academic, industrial, and broader readership, and not raise false hopes for the fast commercial implementation (scientific populism). Overselling of the research results hampers the prospects of other, more balanced studies from landing in the same dissemination channels (journals) or receiving the same credit and distorts the assessment of the novelty and impact by the major stakeholders (funding agencies and general public).

Claims technology	Basic material properties	Conversion factors	Detectors figures of merit	Commercialization feasibility
Direct detectors	X‐ray absorption, mobility, lifetime, resistivity	X‐ray sensitivity	DE, NED, E_1/2_, speed, spatial resolution, DQE, n_1_	Stability, integration with electronics, toxicity, prices
Scintillators	X‐ray absorption, optical Stokes shift, quantum yield	Light yield		
Do not yet claim your paper	X‐ray detection ability	X‐ray detector performance	Commercial implementation	–
May be claimed	X‐ray detection promise	X‐ray detection ability	X‐ray detector performance	Commercial implementation
1	2	3‐4	5‐9	TRL

## 2 General Approach Toward High‐Quality X‐Ray Medical Imaging at Low Doses

**Figure 1:**
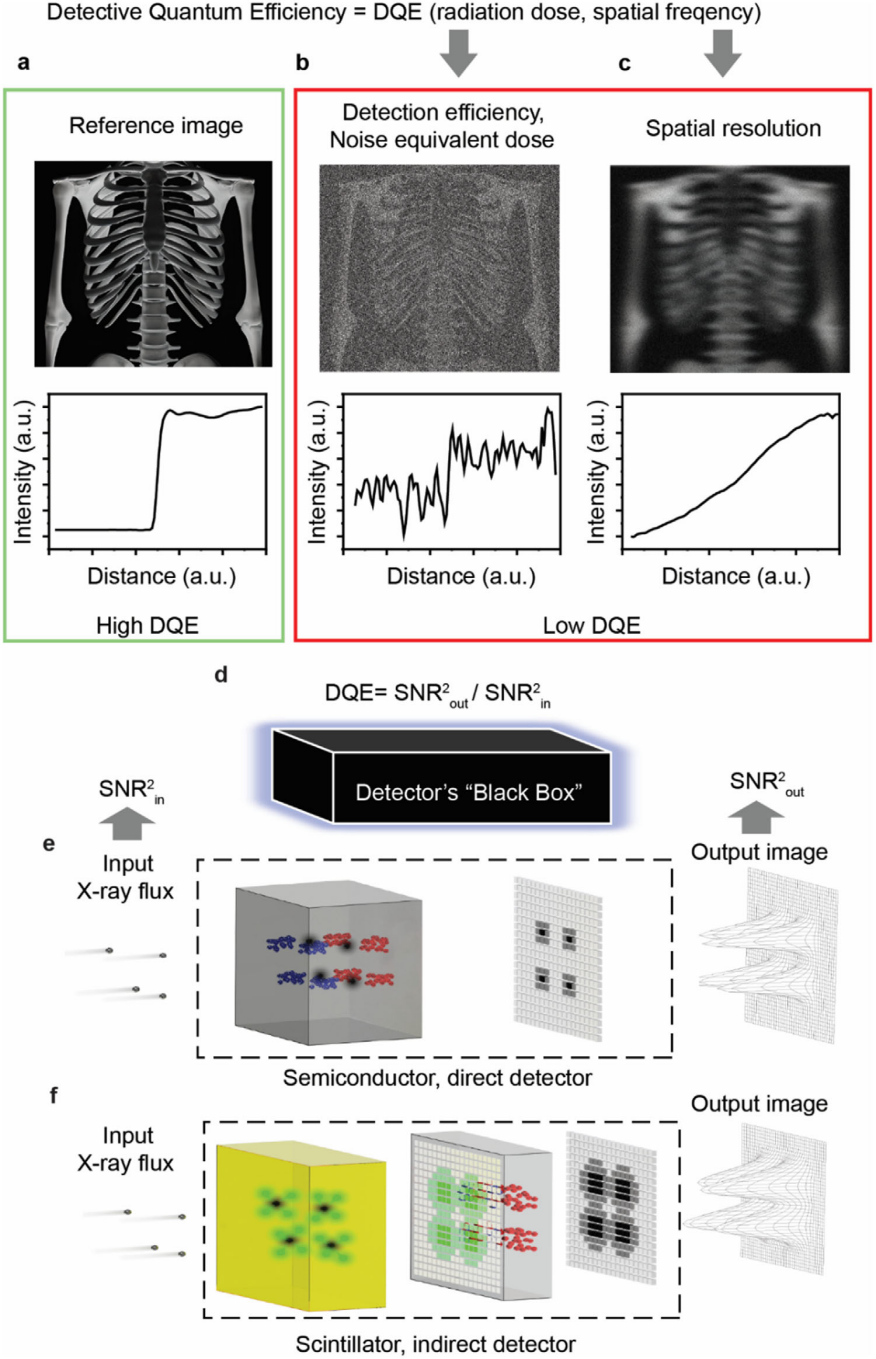
DQE concept. a) Reference high DQE example: high‐quality X‐ray image captured using a detector with high DQE. The graph below the image illustrates the high contrast at the object's edge. b,c) Low DQE examples: degraded X‐ray image quality and corresponding edge contrasts (graphs below the images), caused by low DQE. Specifically, elevated noise (b) due to a high Noise Equivalent Dose and reduced detection efficiency, and image blurring (c) arising from low spatial resolution. The equation above (a–c) describes DQE as a function of dose and spatial resolution. d) Detector's “Black Box” concept alongside DQE definition. e,f) X‐ray imaging detector structure and conversion stages for two major X‐ray detection technologies: direct with semiconductors (f), indirect with scintillators (e).

X‐ray imaging is based on the advantage of high‐energy photons in the deep and density‐specific penetration, enabling visualization of inner object structures with a high spatial resolution. However, high‐energy photons ionize the matter, which in the case of medical imaging poses health risks for patients, proportional to *D*, making the minimization of radiation exposure a major concern. Consequently, achieving optimal imaging quality at the lowest feasible radiation dose (in line with the "as low as reasonably achievable" principle)^[^ ^28^ ^]^ becomes critical. At the same time, the quantum nature of photons ultimately limits how much the dose can be reduced: the maximum possible signal‐to‐noise ratio (SNR) for a given number of photons is defined by their Poisson statistics. For example, to achieve SNR of 10, at least 100 photons per imager pixel are necessary. In practice, the actual *D* required for acceptable image quality exceeds what Poisson statistics alone would predict, particularly due to imperfections in the X‐ray imaging system, which is defined by DQE:^[^ ^16a^ ^]^

(1) $$\mathrm{DQE}\left(n_{\mathrm{photon}/\mathrm{pixel}},\;f\right)=\frac{{\mathrm{SNR}}_{\mathrm{out}}^{2}}{n_{\mathrm{photon}/\mathrm{pixel}}}=\frac{{\mathrm{SNR}}_{\mathrm{out}}^{2}}{{\mathrm{SNR}}_{\mathrm{in}}^{2}}$$

where n_photon/pixel_ is the number of X‐ray photons per imager pixel and is proportional to *D*, SNR_out_ is the actual signal‐to‐noise ratio at detector output, SNR_in_ is the inherent signal‐to‐noise ratio, determined by the Poisson statistic (SNR^2^_input_ = n_photon/pixel_), *f* is the spatial frequency. The DQE measures how much extra exposure is needed compared to the ideal, Poisson statistics‐limited case (in which DQE = 1). A higher DQE indicates that less dose is required to achieve a given image quality, thus directly benefiting patient safety. Importantly, DQE is not a scalar value but a function dependent on dose (considering also X‐ray photon energy) and spatial frequency. The complete characterization of DQE requires evaluating several core factors, including the detector's detection efficiency, the noise‐equivalent dose (**Figure** **1**a,b), and its spatial resolution (Figure 1a,c). By treating the imaging system as a “black box” (Figure 1d), the DQE framework can be universally applied to virtually any type of detector, whether it relies on direct (Figure 1e) or indirect scintillator‐based conversion (Figure 1f). Therefore, we summarize that the DQE metrics are the central figure of merit of X‐ray detectors for low‐dose X‐ray imaging. In the following sections, we review the methods for the evaluation of DQE defining parameters, and we analyze the inconsistencies in common benchmarking approaches in materials research.

## 3 Critical Analysis of Widespread Figures of Merit in X‐Ray Detectors

Objective factors, including the difficulty of accurately assessing DQE, especially for early‐stage detectors, and limited technical expertise within many materials research groups, have led to the widespread adoption of surrogate figures of merit for benchmarking emerging detector materials: X‐ray sensitivity and LoD. While these metrics are attractive due to their simplicity and intuitive measurement procedures, reliance on them can inadvertently emphasize device characteristics that negatively impact overall detector performance, as demonstrated in the subsequent paragraphs.

**Figure 2:**
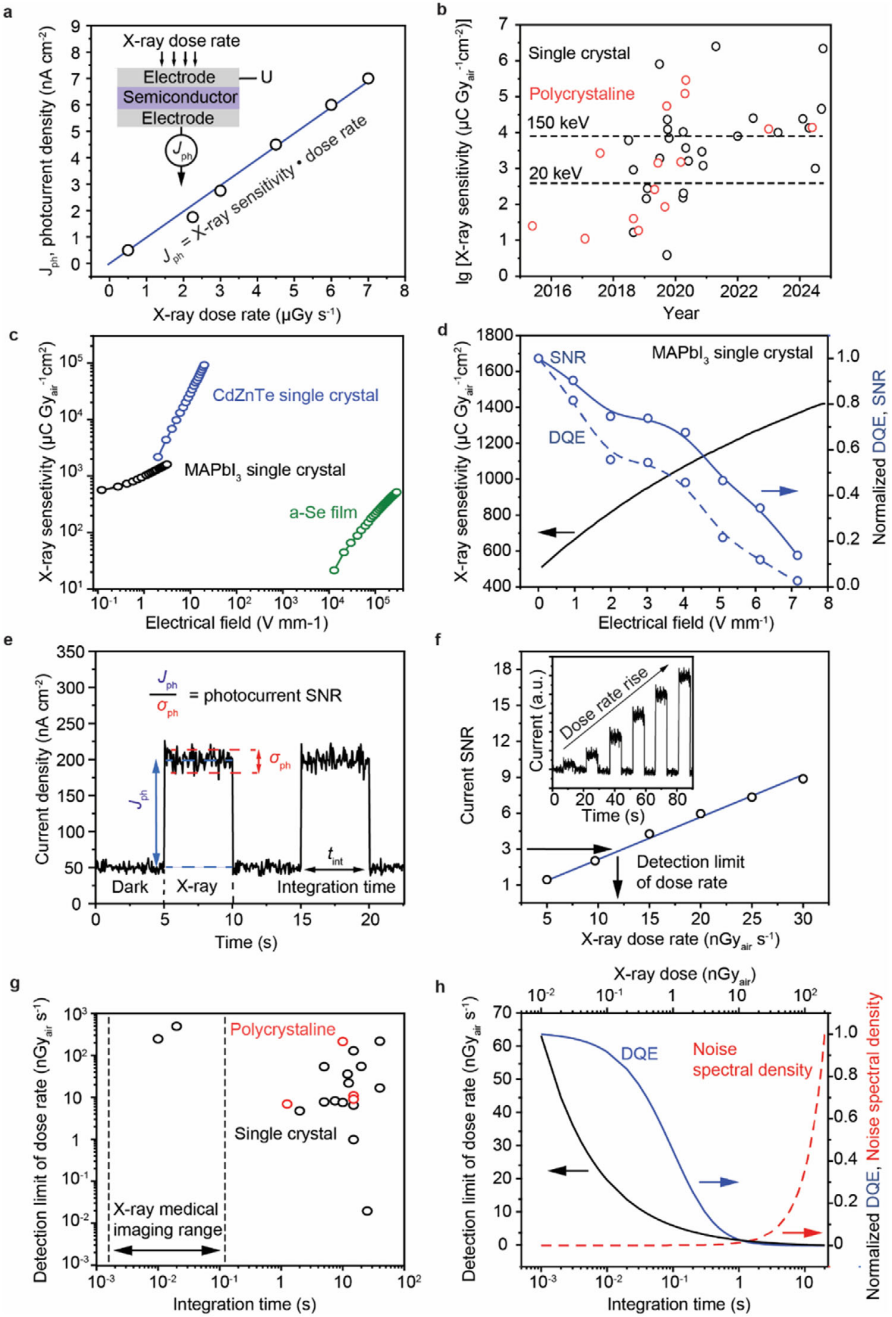
Widespread X‐ray detectors' figures of merit. a) A typical dependence of photocurrent density on dose rate for a direct‐conversion detector (schematic shown in the inset), illustrating the method commonly used to determine X‐ray sensitivity. b) X‐ray sensitivities of perovskite detectors reported across various studies^[^ ^29^, ^35^, ^36^, ^37^, ^38^, ^39^, ^40^, ^41^, ^42^, ^43^, ^44^, ^45^, ^46^ ^]^ over the years. The dashed lines represent theoretical sensitivity limits calculated at different X‐ray energies for a semiconductor with a bandgap of 1.5 eV, assuming complete X‐ray absorption and full charge extraction.^[^ ^47^ ^]^ c) X‐ray sensitivity dependencies on the electrical field for various materials. Imbalanced transport results in photoconductive gain, thus continuous growth of sensitivity without reaching a plateau. Reproduced under the terms of the CC‐BY license.^[^ ^48^ ^]^ Copyright 2023, The Authors. Published by Springer Nature. d) Dependence of X‐ray sensitivity (black solid line), normalized SNR (blue solid line), and DQE (blue dashed line) of a MAPbI_3_ single crystal on the applied electric field, obtained based on data from ref. [48]. e) Typical photocurrent time traces illustrating a series of X‐ray pulses used to determine the current SNR. f) Typical dependence of current SNR on X‐ray dose rate, calculated from the series of X‐ray pulses shown in the inset. Such measurements are commonly employed for estimating LoD. g) LoD values reported in various studies^[^ ^15^, ^32^, ^36^, ^37^, ^38^, ^39^, ^40^, ^41^, ^42^, ^43^, ^44^, ^45^, ^46^, ^49^, ^50^ ^]^ as a function of integration time. h) Typical dependencies of LoD, DQE, and noise spectral density on integration time.

X‐ray sensitivity is typically defined (**Figure** **2**a) as the proportionality factor between X‐ray dose rate $\left(\dot{D}\right)$ and X‐ray induced photocurrent (*J*_ph_):

(2) $$J_{\mathrm{ph}}=X-\mathrm{sensitivity}\cdot \dot{D}$$

Higher X‐ray sensitivity provides a higher photocurrent at the same dose rate, which is often misinterpreted as yielding higher SNR. This perception has sparked a recent race toward ever‐higher X‐ray sensitivities within the metal halide perovskites research community,^[^ ^29^ ^]^ resulting in reported values increasing by orders of magnitude over recent years (Figure 2b). Many reported sensitivity values substantially exceed the theoretical limits^[^ ^30^ ^]^ within the whole X‐ray photon energy range relevant for medical imaging (dashed lines in Figure 2b). Sensitivities surpassing the theoretical limit are typically achieved through photoconductive gain,^[^ ^31^ ^]^ which allows to amplify (until the material's electrical breakdown) the device photocurrent with higher electric fields (Figure 2c). However, this enhancement comes at the expense of significantly increased noise, resulting in substantial degradation of SNR and DQE (Figure 2d). Consequently, the pursuit of excessively high sensitivity conflicts with the fundamental principles of high‐quality medical imaging outlined in Section 1.

Another widely adopted benchmarking metric is the LoD, frequently used to showcase a low‐dose detector's performance. The LoD evaluation is based on the photocurrent SNR (Figure 2e), which is measured as the photocurrent ratio to the current root‐mean‐square (RMS) fluctuation during the defined integration time (*t*_int_) at various dose rates (inset in Figure 2f). The dose rate corresponding to a photocurrent SNR of 3 is designated as the LoD. Although many studies,^[^ ^15^, ^32^ ^]^ justifying the LoD approach cite the International Union of Pure and Applied Chemistry protocols,^[^ ^33^ ^]^ which were originally developed for determining radioactive analyte concentrations and have no relevance to medical imaging detectors. Nevertheless, LoD remains widely popular, often measured without adequate consideration of integration time duration. In most reported cases, it extends into the seconds range (Figure 2g), far longer than the practical medical imaging demands of tens of milliseconds. The drive toward longer integration times artificially improves the photocurrent SNR. However, it increases the contribution of 1/f noise at low frequencies, thus decreasing the charge SNR (Figure 2h), which is more relevant for medical imaging (discussed in more detail in Sections 3 and 6). Consequently, while extending integration times lowers the reported LoD, it simultaneously degrades the DQE, underscoring how reliance on LoD as a performance metric can mislead researchers and hinder real progress toward achieving effective low‐dose X‐ray imaging.

In addition to misconceptions prevalent in direct‐conversion detector research, studies on novel scintillator materials also commonly suffer from incomplete performance reporting. Most reports focus primarily on parameters such as light yield and, at best, spatial resolution, while frequently omitting DQE, which is a standard benchmarking metric for commercially available scintillator medical imaging detectors. Furthermore, recent efforts in novel scintillator development emphasize achieving high spatial resolution through thin‐film scintillator layers,^[^ ^3^, ^34^ ^]^ often disregarding their inherently reduced X‐ray absorption efficiency. This approach contradicts the primary goal of low‐dose X‐ray imaging, regardless of how high the scintillator's light yield may be. Therefore, comprehensive reporting of both spatial resolution and absorption efficiency, ideally integrated through DQE, is essential for an accurate and fair assessment of new scintillation materials intended for medical X‐ray imaging, as shown in the paragraphs below.

## 4 Signal and Noise Formation

**Figure 3:**
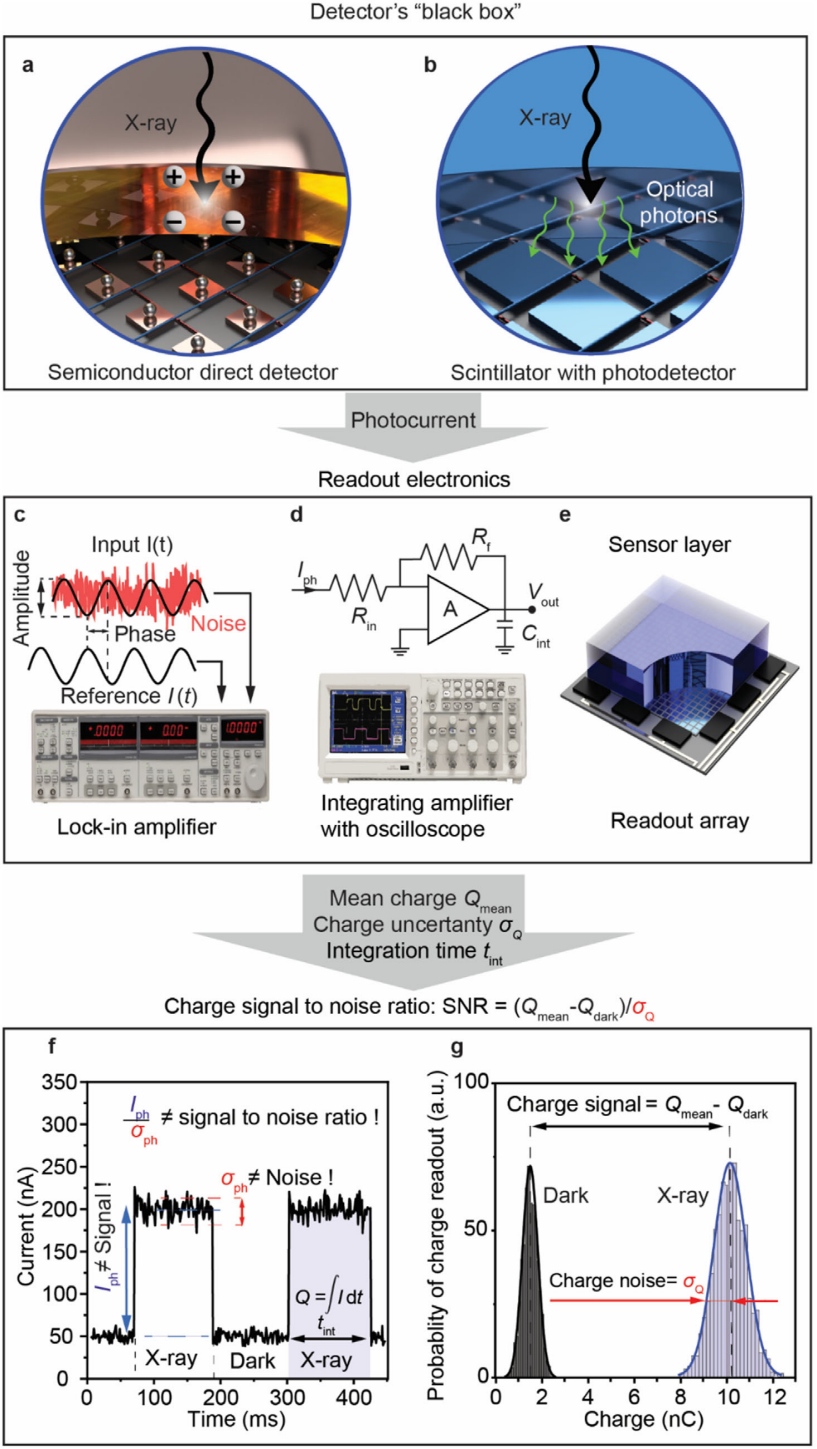
Signal processing. a,b) Detector “black box,” illustrating the conversion of X‐ray photons into photocurrent for semiconductor direct‐conversion detectors (a), and into optical photons within a scintillator, followed by subsequent conversion into photocurrent using a photodetector (b). c–e) Examples of readout electronics: lock‐in amplifier (c), integrating amplifier with oscilloscope (d), readout array (e). f) Representative photocurrent time traces demonstrating a typical series of X‐ray pulses used for charge collection measurements. g) Typical distributions of measured charges under dark conditions and X‐ray irradiation, highlighting definitions of charge signal and noise.

To establish correct metrics, relevant for low‐dose, high‐performing detectors, i.e. with high DQE, it is important to review the sequence of physical phenomena forming signal and noise. According to applied linear‐systems theory,^[^ ^16a^ ^]^ both signal and noise at the detector input propagate linearly through the detector system, i.e. the X‐ray photon flux and its associated noise (determined by Poisson photon statistics) are directly transferred into the detector response, measured as photocurrent. From this perspective, a detector system may be simplified to a concept of a “black box” that converts incoming X‐ray photons into photocurrent. This unifies various detectors architectures, including semiconductor detectors, where the photocurrent is generated directly (**Figure** **3**a), as well as scintillator‐based detectors, where X‐ray photons are first converted into optical photons, which subsequently generate photocurrent in the photodetector (Figure 3b). Subsequently, the generated photocurrent is read out by electronics, the configuration of which depends on the detector architecture and the development stage. At the early research phase, single‐channel detector prototypes are typically coupled either to lock‐in amplifiers^[^ ^1^, ^48^ ^]^ (Figure 3c) or integrating amplifiers coupled with oscilloscopes^[^ ^51^ ^]^ (Figure 3d). At more advanced stages of development, detectors are integrated with multi‐pixel readout systems (Figure 3e), which may employ thin‐film transistors,^[^ ^3^ ^]^ charge amplifiers,^[^ ^52^ ^]^ or photodiodes.^[^ ^6^ ^]^ Regardless of specific architecture, the readout electronics measure the collected charge (*Q)* by integrating the current during *t*_int_ each exposure cycle (Figure 3f). The noise corresponds to the uncertainty in a charge measurement, represented by the RMS deviation (*σ_Q_*) of the mean collected charge (*Q*_mean_). Here we consider that environmental noise (e.g., electromagnetic interference, mechanical vibrations and etc.) is minimized through proper shielding of the setup and thus can be neglected. The signal is then defined as the difference between *Q*_mean_ under X‐ray exposure and the mean charge measured under dark conditions, *Q*_dark_ (Figure 3g). Consequently, the charge SNR is defined as:

(3) $$\mathrm{Charge}\;\mathrm{SNR}=\frac{Q_{\mathrm{mean}}-Q_{\mathrm{dark}}}{\sigma_{Q}}$$

This definition remains valid regardless of the detection mode (whether charge‐integration or photon‐counting) and the measurement units (proportional to the collected charge, such as analogue‐to‐digital converter units or photon‐counting events). It is particularly important to utilize the charge SNR for characterizing detector performance, as it directly reflects the accuracy of dose measurement, which is the most relevant to medical imaging, rather than the current SNR, which represents the accuracy of dose rate estimation. Clearly distinguishing between dose and dose rate accuracy is essential, even though these concepts might intuitively appear similar when integration time is not explicitly considered. The integration time significantly affects RMS uncertainties for dose and dose rate measurements, but in opposite ways. Specifically, longer *t*_int_ leads to an increase in RMS in dose (i.e. charge signal) measurements, while simultaneously reducing uncertainty in dose rate (i.e. current signal) measurements, as discussed further in Section 6. To note, strictly speaking, the uncertainty from dark measurements should also be included in Equation (4). However, in practice, this uncertainty is typically negligible compared to that obtained under X‐ray exposure because dark measurements can be extended as necessary to achieve the desired accuracy. In contrast, the duration of X‐ray exposure measurements must remain short to reflect clinically relevant conditions.

## 5 Characterization of Signal and Noise as Functions of Radiation Dose ‐ Detection Efficiency and Noise Equivalent Dose

### 5.1 Ionizing Radiation Dose

The radiation dose, *D* can be described as the energy absorbed per mass unit (J∙kg^−1^ or Gray) or as a number of X‐ray photons incident upon the detector pixel area or total detector area in case of single‐readout channel (*n*_photon/pixel_). For a homogenous, parallel X‐ray beam with the direction perpendicular to the detector area *A* and *n*_photon/pixel_ are related as (it can be derived from equation 3.17 in ref. [53]).

(4) $$D\;[Gy_{\mathrm{air}}]=\frac{\;{\left(\frac{\mu }{\rho }\right)}_{\mathrm{air}}E_{\mathrm{ph}}\;n_{\mathrm{photon}/\mathrm{pixel}}}{A}$$

where *E*_ph_ is the X‐ray photon energy. It should be noted that this formula is applicable only for a mono‐energetic or quasi mono‐energetic (filtered to a narrow band spectrum) photon flux incident normal to the detector surface. In a more general case, an X‐ray source may have a broad energy spectrum. Thus, an averaging of ${\left(\frac{\mu }{\rho }\right)}_{\mathrm{air}}\cdot E_{\mathrm{ph}}$ product over the X‐ray source spectrum is necessary. Equation (4) is applied only for the absorbed dose in air (not for the dose in the sensor material!), since for its derivation the charge particle equilibrium is assumed ‐ the dose build‐up region is ignored, and all secondary photons escape the interaction volume. Equation (4) is still of practical value, since radiation diagnostics dosimeters commonly measure air kerma.

### 5.2 Signal Dependence on Dose

The relationship between signal and dose is linear within a certain dose range, provided the signal is significantly above the noise threshold (Figure S1a, Supporting Information):

(5) $$\mathrm{Signal}\left(D\right)=\mathrm{DE}\cdot \alpha \cdot G\cdot D$$

where DE (Detection Efficiency) is defined as the ratio of detected photons to incoming ones. DE is a measure of the signal conversion efficiency. For charge‐integration mode, it incorporates both X‐ray absorption efficiency and the collection efficiency of the generated charge (in direct‐conversion detectors) or emitted optical photons (in scintillator‐based detectors). For scintillator detectors, it also accounts for the scintillator's form factor, reflectance, refraction properties, and the coupling efficiency to the photodetector and its quantum efficiency within the scintillator's emission wavelength range. Wherein, for photon‐counting mode, DE is mainly determined by X‐ray absorption efficiency. The parameter *α* is a conversion coefficient that describes the transformation of the dose (i.e., X‐ray photon number) into charges (i.e., X‐ray sensitivity for direct conversion) or optical photons (i.e., light yield for scintillators) for charge‐integration mode, or into counts for photon‐counting mode. For instance, for a direct‐conversion semiconductor detector, *α* is equal to the product of detector area (*A*) and the theoretical X‐ray sensitivity of the photodiode calculated according to Equation (4). This theoretical sensitivity can be derived using Equations (15) and (17) from ref. [30] and assuming the Klein rule for electron‐hole pair creation:^[^ ^54^ ^]^

(6) $$X-\mathrm{ray}\;\mathrm{sensitivity}=C\frac{e}{\;{\left(\frac{\mu }{\rho }\right)}_{\mathrm{air}}W_{\pm }}$$

where *e* is the elementary charge, *W*_±_ is the electron‐hole pair creation energy by ionization,^[^ ^54^ ^]^ $\;\;{\left(\frac{\mu }{\rho }\right)}_{\mathrm{air}}$ – X‐ray mass energy absorption coefficient in the air (X‐ray energy dependent), C is the constant dependent on the units used. For example, if X‐ray sensitivity is expressed in units of µC cm^−2^ Gy^−1^_air_, *W*_±_ units are eV, ${\left(\frac{\mu }{\rho }\right)}_{\mathrm{air}}$ units are cm^2^ g^−1^, then C is equal to 10^3^ µC eV g^−1^ Gy^−1^_air_ *e* ^−1^_._ Important to note, it might be complicated to evaluate the theoretical X‐ray sensitivity of novel semiconductors using Equation (6), since while *W*_±_ parameter follows Klein's rule^[^ ^54^ ^]^ for most semiconductors there are several exceptions. Thus, Equation (6) should be used with caution when *W*_±_ is unknown. X‐ray mass energy absorption coefficient is used specifically for the air (not for the detector sensor material!), since most dosimeters measure air kerma, which is equivalent to the absorbed dose in air for X‐ray energies relevant to X‐ray medical imaging applications. Also, Equation (6) assumes monoenergetic X‐ray photons. In the general case of a polyenergetic photon flux, X‐ray sensitivity has to be averaged over the spectrum, considering the dependence of $\;{\left(\frac{\mu }{\rho }\right)}_{air}$ on X‐ray energy. For materials exhibiting near‐100% charge collection efficiency, for instance, CdTe with the bandgap of 1.5 eV, the ultimate X‐ray sensitivities, according to Equation (6), would approach 3500 µC·cm^−2^·Gy^−1^_air_ for 40 keV X‐ray energy. The units of *α* are dependent on the dimensions in which the signal and the dose are expressed (e.g., three pairs of axes in Figure S1a, Supporting Information). Independent evaluation of *α *can be complex, particularly for scintillators, because their light yield may vary with X‐ray energy^[^ ^55^ ^]^ and can be quenched at high doses. The parameter *G* is an electronic gain of the detector, which may include a photoconductive gain of an active material for the direct‐conversion materials.

For the case when Equation (5) is applicable and the signal vs dose dependence is experimentally measured, it is possible to determine the entire DE∙*α∙G* product. Estimating each parameter separately might require a dedicated experiment. If the detector system is able to count single X‐ray photons (corresponds to 1 count/(X‐ray photon), *G * =  1, case A in **Table** **2**) or it is working in a charge‐integration mode with specific *G* (case B in Table 2), then the product DE∙*α∙G* value (obtained from Figure S1a, Supporting Information) may be used for direct determination of DE:

(7) $$\mathrm{DE}=\frac{n_{\mathrm{photon}/\mathrm{pixel}\_\mathrm{detected}}}{n_{\mathrm{photon}/\mathrm{pixel}\_\mathrm{incoming}}}=\frac{\mathrm{Signal}\left(D\right)}{\alpha \cdot G\cdot D}$$

**Table 2:** Characterization checklist. This table summarizes key figures of merit for both single‐readout‐channel and array detectors. For array detectors, metrics such as NED, DE, E50 DQE versus dose, and speed should be given as pixel‐wise statistical distributions, with the mean value reported as the representative figure.

Applicable architecture	Figure of merit^a)^	Estimation steps
Single‐readout channel and array detectors	Noise equivalent dose: NED	1) Determine the slope from the signal vs dose dependence (DE∙*G*∙*α* product at Figure S1a, Supporting Information). 2) Using the offset and the slope from the noise vs dose dependence (Figure S1c, Supporting Information) and determined before DE∙*G*∙*α* product, estimate NED both in dose and photon number units. 3) Using Equation (14), plot NED dependence on X‐ray energy (Figure S2a, Supporting Information).
	Detection efficiency: DE	*Case A ‐ photon‐counting detection mode*. Determine directly from the slope of the signal vs dose dependence (DE∙*G*∙*α* product at Figure S1a, Supporting Information), as in this case *G*∙*α* = 1 count/photon. *Case B – charge‐integration mode, when G*∙*α product is known*. Determine directly from the slope of the signal vs dose dependence (DE∙*G*∙*α* product at Figure S1a, Supporting Information). *Case C – charge‐integration mode, when G*∙*α is not known*. Determine the slope from the signal vs dose dependence (DE∙*G*∙*α* product at Figure S1a, Supporting Information). Measure the noise vs dose dependence (Figure S1c, Supporting Information). Using DE∙*G*∙*α* product to express noise in photon‐equivalent units. Determine DE from the fit by Equation (11) of the noise vs. dose dependence. *Case D – quick evaluation for any detection mode*. The upper limit of detection efficiency can be determined as equal to the absorption efficiency (Equation 13)
	X‐ray energy at 50% absorption efficiency: *E*_50_	Determine from X‐ray absorption efficiency dependence on X‐energy (Figure S2a, Supporting Information).
	Detective Quantum Efficiency dependence on dose and X‐ray energy: DQE(*D*, *E*)	Using previously determined DE, NED, and X‐ray absorption efficiency, plot DQE vs dose and energy (Figures S1f and S2a, Supporting Information). The latter is applicable only using (quasi) monoenergetic X‐ray spectrum. Otherwise, report the used X‐ray beam quality only.
	Rise and fall time, cut‐off frequency: τ_r_, τ_f_, *f*_3dB_.	*Case I ‐ photon‐counting detection mode*. Determine the detector speed from the rise time of a single‐photon counting event. Calculate cut‐off frequency using Equation (17). *Case II ‐ charge‐integration mode, when X‐ray is possible to apply as a square pulse*. Determine the detector speed from the slowest of the rise time and the fall time (Figure 4b). Calculate the cut‐off frequency using Equation (17). *Case III ‐ charge‐integration mode, when variation of X‐ray modulation frequency is possible* Determine the cut‐off 3dB frequency of the signal dependence on frequency (Figure 4d), and calculate response time through Equation (17).
Array detectors	Spatial resolution: *f_20_*	Determine the spatial resolution from the Modulation transfer function dependence on spatial frequency (Figure 5c–f).
	Noise power spectrum: NPS(*f*)	Using noise data, calculate NPS vs spatial frequency. (Figure 5h)
	Detective Quantum Efficiency dependence on spatial frequency: DQE (*f*)	Using MTF, NPS vs dependencies, and Equation (21), plot DQE vs spatial frequency.
	Number of photons passing through the smallest resolvable feature to achieve SNR=1: *n*_1_	Using all previous metrics, determine *n*_1_ according to Equations (32) and (33); or determine from images of the liming spatial resolution feature *vs*. dose (Figure 5k,m).

^a)^ written in the format “full figure of merit name: abbreviation”.

When the parameters *G* or α are challenging to estimate, the DE can be more reliably evaluated using noise analysis methods, as discussed in subsequent sections. Figure S1b (Supporting Information) provides examples of signal‐dose relationships for various detector materials, explicitly indicating DE where determination by the detector system is feasible.

### 5.3 Noise Dependence on Dose

Noise is the RMS or standard deviation of the mean signal value. Square of total noise (${\mathrm{Noise}}_{T}^{2}$) is linearly dependent (Figure S1c, Supporting Information) on a photon number *n*_photon/pixel_ according to (for simplicity, *α* is expressed as arbitrary units (a.u.) per photon):

(8) $${\mathrm{Noise}}_{T}^{2}\left[{\left(a.u.\right)}^{2}\right]=\mathrm{DE}\cdot G^{2}\cdot \alpha^{2}\cdot n{\mathrm{photon}/\mathrm{pixel}+\mathrm{Noise}}_{D}^{2}$$

We should note that the described approach isn't applicable if at least one of the dependencies, Equations (5) and (8), is substantially nonlinear. According to the definition,^[^ ^56^ ^]^ NED is the radiation dose that generates the quantum noise, which corresponds to the situation when the two right terms of Equation (7) are equal. The first term is described by Poisson statistics of absorbed X‐ray photons. The second term is the intrinsic noise of the detector (Noise_D_) in the absence of radiation. Thus, NED can be determined as an offset/slope ratio from the linear fit of the experimentally measured dependency N_T_(*D*) in units of either photon number or dose:

(9) $$\mathrm{NED}\left[\mathrm{photon}\right]=\frac{{\mathrm{Noise}}_{D}^{2}\left[{\left(a.u.\right)}^{2}\right]}{\mathrm{DE}\cdot G^{2}\cdot \alpha^{2}}$$

(10) $$\mathrm{NED}\left[Gy_{\mathrm{air}}\right]=\frac{\;{\left(\frac{\mu }{\rho }\right)}_{air}\cdot E_{ph}\cdot \mathrm{NED}\left[\mathrm{photon}\right]}{A}$$

For the determination of NED, it suffices to use the experimentally obtained product DE∙*G* ^2^∙*α* ^2^, while estimation of separate values for DE, *G*, and *α* is not necessary. We should note that Noise_D_ drastically increases with *G*, thus NED does not decrease with increasing *G*. NED in X‐ray photon number units (Equation 9) points to the detector system's ability to count single photons, when NED << 1 photon. Therefore, NED can be interpreted as a measure of noise conversion efficiency. Simultaneously, expressing NED in dose units (Equation 10) is valuable, as it directly characterises detector performance under low‐dose conditions relevant to medical imaging applications. As follows from Equation (10), the NED value is inversely proportional to detector area and might become attractively low for large detectors. While it is appealing for dosimetry, it is, however, not beneficial for medical imaging since it hampers spatial resolution. It is therefore crucial, for medical imaging, that both parameters of NED[Gy_air_] and spatial resolution, or at least pitch size, when spatial resolution is unknown, are reported simultaneously. NED is applicable for both semiconductor and scintillator detectors, as it characterizes the noise of the whole detector assembly.

To determine DE directly from the noise analysis, the dependence Noise_T_ on dose should be represented in photon‐equivalents ‐ a factor that scales the noise amplitude to the signal of a single incident X‐ray photon. Equation (8) should thus be divided by the product (DE∙*G*∙*α*)^2^, known from Equation (5) (Figure S1a, Supporting Information):

(11) $${\mathrm{Noise}}_{T}^{2}\left[{\left(\mathrm{photon}-\mathrm{equvialent}\right)}^{2}\right]=\frac{n_{\mathrm{photon}/\mathrm{pixel}}}{\mathrm{DE}}{+\mathrm{Noise}}_{D}^{2}$$

DE can be extracted as the inverse slope parameter from the linear function fit of noise values (Figure 2d), according to Equation (7) (case C in Table 2). Figure S1d (Supporting Information) illustrates examples of noise dependencies on dose for various detector materials, with corresponding values of NED and DE indicated. The characterization described above applies equally to single‐channel detectors and multi‐pixel array detectors. For the last case, DE and NED should be reported as mean values averaged across all channels. The signal and noise can be determined from a single‐frame acquisition under homogeneous irradiation of the array. Data collected from each array readout channel can then be used to construct the Gaussian distribution illustrated in Figure 3g.

### 5.4 Detective Quantum Efficiency Dependence on Dose

NED and DE can be combined into a single characteristic—DQE (Figure S1e, Supporting Information). The latter is formulated^[^ ^57^ ^]^ as a squared ratio of the output detector signal‐to‐noise, ${\;\mathrm{SNR}}_{\mathrm{out}}^{2}=\frac{{\left(\mathrm{DE}\cdot \alpha \cdot G\cdot n_{\mathrm{photon}/\mathrm{pixel}}\right)}^{2}}{\mathrm{DE}\cdot G^{2}\cdot \alpha^{2}\cdot n_{\mathrm{photon}/\mathrm{pixe}l}{+\mathrm{Noise}}_{D}^{2}}$, as obtained from Equations (5) and (8), to the input (or ideal) signal‐to‐noise ratio, ${\mathrm{SNR}}_{\mathrm{in}}^{2}=n_{ph}$, defined by Poisson statistics of the incident X‐ray photon flux.^[^ ^58^ ^]^ Taking also into account Equations (9) and (10) one obtains:

(12) $$\mathrm{DQE}\;\left(D\right)=\frac{{\mathrm{SNR}}_{\mathrm{out}}^{2}}{{\mathrm{SNR}}_{\mathrm{in}}^{2}}=\frac{\mathrm{DE}}{1+\frac{\mathrm{NED}[\mathrm{photon}]}{n_{\mathrm{photon}/\mathrm{pixel}}}}=\frac{\mathrm{DE}}{1+\frac{\mathrm{NED}[\mathrm{Gy}]}{D\;}}$$

DQE characterises the square of the detector signal‐to‐noise ratio gained per unit of photon quantity, i.e., exposure dose. DQE is commonly studied as a function dependent on the spatial frequency^[^ ^16b^ ^]^ for array detectors, but is a valid characteristic to report also for single readout channel detectors, often used for early‐stage development. Figure S1f (Supporting Information) illustrates representative examples of DQE (*D*) functions for various detector materials.

## 6 Influence of X‐Ray Energy

Typically, an X‐ray tube with a polyenergetic spectrum is used for X‐ray medical imaging. Such a spectrum is characterized by a standard diagnostic beam quality series.^[^ ^59^ ^]^ For the X‐ray detection performance characterization, equivalently valid to use X‐ray spectrum with specific beam quality (close to intended application) or to apply monochromatic or narrow width spectrum with a mean X‐ray photon energy ($\bar{E}$), which could be obtained with a set of attenuators,^[^ ^60^ ^]^ monochromators,^[^ ^61^ ^]^ or by using radioactive gamma‐emitting isotopes. The (quasi)monochromatic X‐ray spectrum can be particularly beneficial for the detector characterization, since it allows evaluating the entire operational X‐ray energy range by extrapolating DQE (*n*_photon/pixel_), NED and DE (Figure S2a, Supporting Information) using the dispersion of X‐ray Absorption Efficiency (AE), (case D in Table 2):

(13) $$\mathrm{AE}\left(E_{ph}\right)=\left(1-\exp\left(-\mu \left(E_{\mathrm{ph}}\right)d\right)\right)$$

where *µ* is the linear absorption coefficient (dependent on *E*_ph_, which can be calculated using the atomic composition and specific mass,^[^ ^62^ ^]^) *d* is the detector thickness. Using Equation (10) and assuming *α ∼ E*_ph_ and DE *∼* AE:

(14) $$\mathrm{NED}\left(E_{\mathrm{ph}}\right)\approx {\left(\frac{\bar{E}}{E_{\mathrm{ph}}}\right)}^{2}\times \frac{\;\mathrm{AE}\left(\bar{E}\right)}{\mathrm{AE}\left(E_{\mathrm{ph}}\right)}\times \mathrm{NED}\left(\bar{E}\right)$$

(15) $$\mathrm{DE}\left(E_{ph}\right)\approx \mathrm{DE}\left(\bar{E}\right)\frac{\mathrm{AE}\left(E_{\mathrm{ph}}\right)}{\mathrm{AE}\left(\bar{E}\right)}$$

(16) $$\mathrm{DQE}\left(E_{ph},n_{ph}\right)\approx \frac{\mathrm{DE}\left(E_{\mathrm{ph}}\right)}{1+\frac{\mathrm{NED}\left(E_{\mathrm{ph}}\right)}{n_{\mathrm{photon}/\mathrm{pixel}}}}$$

The plot of energy‐dependent DQE for different photon numbers shows that at each dose, there is an optimal energy range. At high doses, DQE becomes limited by AE, particularly at higher energies. In this context, a useful metric is also the highest X‐ray energy at which Absorption Efficiency reaches 50% (E_50%_). Notably, the Absorption Efficiency not only directly impacts DE and DQE, but also indirectly influences the detector speed and the maximal counting rate (for photon counting detection mode). Figure S2b–e (Supporting Information) illustrates examples of AE, NED, DE, and DQE, respectively, dependencies on X‐ray energy, calculated for various detector materials.

Overall, for a comprehensive characterization of a single‐readout‐channel X‐ray detector, we recommend reporting coherently following metrics (Table 2): E_50%_, DE, NED (in both units: dose and photon number), stating the X‐ray energy (or X‐ray spectrum) at which both DE and NED were measured.

## 7 Temporal Response

### 7.1 Influence of Temporal Response on Signal and Noise Characteristics

**Figure 4:**
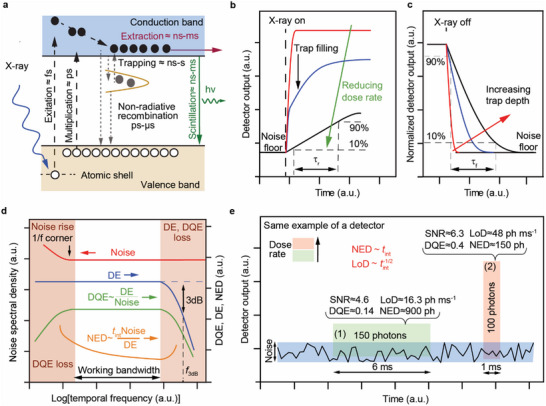
Temporal response in radiation detectors. a) Schematic band structure of a direct and indirect conversion semiconductor detector. X‐ray charge carrier's excitation, multiplication, trapping, non‐radiative recombination, scintillation, and extraction are illustrated alongside corresponding time ranges. b,c) Effect of charge trapping on the detector response rise time (b) and fall time (c). d) Generic frequency dependencies of the noise spectral density, DE, DQE and NED. e) Illustration of the effect of various integration times and X‐ray doses on detector performance metrics (DQE, SNR, NED and LoD), emphasizing LoD as a rather irrelevant characteristic for assessing the utility of the detector for low‐dose imaging, as better SNR, DQE and NED are obtained with the smaller number of X‐ray photons with the detector with improved working bandwidth (lower *t_int_*).

With sufficiently fast readout electronics, the temporal response of an X‐ray detector is limited by the charge carrier recombination dynamics and the charge transport in a sensor material. Both in direct detection with semiconductors and in scintillator materials, an X‐ray photon initially excites an electron from an inner atomic shell to a high level of a conductive band (via photoelectric effect or Compton scattering, **Figure** **4**a). This hot electron then ionizes the sensor material, producing a multitude of high‐energy electrons, which then thermalize in the conduction band. Typically, the timescales of the excitation and multiplication are in the range of femtoseconds and picoseconds, respectively, and as such, do not substantially contribute to the temporal characteristics of the detector. On the other hand, decisive are slower (i.e., nanoseconds to seconds), material‐specific processes: extraction of charge carriers (for semiconductors) or photoluminescence (for scintillators), along with the carrier trapping and non‐radiative recombination (Figure 4a).

The intrinsic temporal response of the detector (without considering any possible instrumental effects like jitter, time walk, etc.) can be expressed as the rise and fall times, τ_r_ and τ_f_, of the detector signal amplitude response between 10% and 90%^[^ ^63^ ^]^ under a square‐shaped X‐ray pulse (Figure 4b,c, case II in Table 2) or a single‐photon counting event (case I in Table 2). The steep, fast component of the rise or fall time is either 1) the transient time in direct‐conversion detectors, the time for collecting the drifting charges, or 2) the luminescence lifetime arising from the radiative recombination in scintillators. The subsequent slower component, if any, usually arises from the initially trapped and subsequently released charges that are then collected at the electrodes or, in scintillator, give rise to the delayed luminescence. Charge trapping may have a complex effect. The signal rise and fall traces become slower for higher trap densities and their greater depth (Figure 4c). Furthermore, a dependence of the response time on X‐ray dose rate might emerge, ultimately yielding faster apparent detector response at high dose rates when the traps are filled (Figure 4b). It is thus important to measure temporal characteristics at practically relevant doses and dose rates. Here, a typical flaw would be to report, for instance, DE at a low dose rate, while the detector speed is presented for a high dose rate.

The detector time response τ is the slowest value of either τ_r_ or τ_f_. The temporal frequency at which DE drops by 3 dB is defined as a cut‐off frequency *f*_3dB_, referred to as detector bandwidth:^[^ ^64^ ^]^

(17) $$f_{3\mathrm{dB}}=\frac{\ln\left(9\right)}{2\pi \tau }\approx \frac{0.35}{\tau }$$

Notably, besides the DQE roll‐off at high frequencies (Figure 4d), there is also the DQE drop at frequencies lower than the so‐called “1/f noise corner” (typical in sub‐Hz range) due to a significant noise rise (Figure 4d). The frequency range optimal for DQE, i.e., working bandwidth *Δf*, can thus be defined, which is located between the 1/f noise corner and the *f*_3dB_ DE cut‐off (Figure 4d, case III in Table 2). Within *Δf*, the intrinsic detector noise (Noise_D_) chiefly originates from the Jonson‐Nyquist thermal noise and the dark current electron shot noise, and the noise current *I_n_* is proportional to the square root of the chosen frequency bandwidth *B*.^[^ ^65^, ^66^ ^]^ Thus, integrated noise charge, i.e., Noise_D_, is proportional to the square root of the integration time:^[^ ^48^, ^67^ ^]^

(18) $$\mathrm{Nois}e_{D}=\int_{0}^{t_{\mathrm{int}}}I_{n}dt∼\int_{0}^{t_{\mathrm{int}}}\sqrt{B}dt∼\sqrt{t_{\mathrm{int}}}$$

assuming *B *= 0.5t_int_^−1^ according to the Nyquist‐Shannon theorem. Equation (18) yields NED ∝ *t_int_* (using Equation 9), and, given that the integrated signal ∝ *t*_int_, one obtains SNR ∝$\;\sqrt{t_{\mathrm{int}}}$. The latter scaling is valid for doses that are low enough, i.e., comparable to NED, when Noise_D_ is comparable to photon shot noise (first term in Equation 8).

In the recent literature, a common strive is to report the lowest detectable dose rate, also known as the lowest limit of dose rate detection, LoD, which is a dose rate for achieving SNR_LoD_ = 3. However, we now show that LoD is, at best, a useless figure of merit since the condition of SNR = 3 may be achieved for an arbitrary dose rate if suitably long *t*_int_ is taken in the experiment (considering SNR_out_ ∝$\;\sqrt{t_{\mathrm{int}}}$). Alternatively, and commonly, LoD is calculated using linear extrapolation of the applied dose rate $\dot{D}$ at the measured SNR_out_ down to SNR_LoD_ = 3 (assuming SNR ∝ dose rate):^[^ ^48^ ^]^

(19) $$\begin{array}{ccc} \mathrm{LoD} & = & \frac{\mathrm{SN}R_{\mathrm{LoD}}}{\mathrm{SN}R_{\mathrm{out}}}\dot{D}\\ & = & \frac{3}{\mathrm{SN}R_{\mathrm{out}\;}}\dot{D}∼\left|\dot{D}=\mathrm{const},\mathrm{SN}R_{\mathrm{out}}∼\sqrt{t_{\mathrm{int}}}\;\;\right|∼\frac{1}{\sqrt{t_{\mathrm{int}}}} \end{array}$$

where one sees that LoD is inversely proportional to *t*_int_ under the constant dose rate. Thus, we recommend excluding LoD from reporting whatsoever, because the pursuit of the lowest LoD motivates experiments with unreasonably long *t*_int_ and hence higher acquired dose, showing the absurdity of LoD for the assessment of the materials for low‐dose imaging, as illustrated in Figure 4e. The cases (1) and (2) differ in the obtained LoD. Case (1) depicts a low dose rate and longer integration time, yielding lower LoD, but higher accumulated dose compared to case (2). Most importantly, in case (2), employing a higher dose rate and much shorter integration time, one receives improvement on all characteristics relevant for low‐dose imaging: DQE and SNR are increased, while NED is reduced. Instead of striving for the smallest LoD, one must optimize for the lowest NED, whose frequency dependence will determine the optimal *t_int_* for a given detector. Specifically, the shortest signal integration time *t*_int_ to be chosen is determined by the working bandwidth,^[^ ^67^ ^]^ i.e., the DQE high‐frequency cut‐off, discussed above, at which also the NED reaches the minimum (Figure 4d):

(20) $$t_{int}=\frac{1}{2\Delta f}$$

Overall, for comprehensive characterization of a temporal response from an X‐ray detector, we recommend reporting coherently the following metrics (Table 2): τ_r_, τ_f_, *f*_3dB_. We encourage the measurements of NED and DE to be taken with the fastest possible integration time, determined by the working bandwidth, which is limited either by the detector or by the application requirements. For example, photon counting detectors must exhibit response times in the sub‐ns range for the rise time in positron emission tomography,^[^ ^68^ ^]^ tens of nanoseconds for rise/fall time in computed tomography,^[^ ^69^ ^]^ and µs‐range in mammography and radiography.^[^ ^48^, ^70^ ^]^ With charge‐integration detectors, the response time is set by the X‐ray tube pulse duration, which is 3‐20 milliseconds for modern medical imaging devices.^[^ ^71^ ^]^

### 7.2 Characterization Methods of Detector Temporal Response

To accurately evaluate a detector's speed limit, the excitation pulse duration from the source must be shorter than the detector's response time. For single‐photon counting detectors, appropriate excitation methods include X‐ray tubes operated at sufficiently low flux^[^ ^72^ ^]^ (Figure S3a, Supporting Information) or gamma‐ray emitting radioactive isotopes^[^ ^73^ ^]^ (Figure S3b, Supporting Information), both of which generate discrete single‐photon pulses at the detector. For charge‐integration detectors, suitable excitation methods include laser pulses^[^ ^74^ ^]^ (Figure S3c, Supporting Information), X‐ray tube intensity modulation via a rotating thick X‐ray opaque chopper wheel^[^ ^2^, ^48^ ^]^ (Figure S3d, Supporting Information), or laser‐triggered pulsed X‐ray tubes^[^ ^75^ ^]^ (Figure S3e, Supporting Information). These excitation techniques can be applied regardless of the detector material type, whether semiconductor‐based or scintillator‐based. Notably, intrinsic physical limits on response speed differ significantly between these detector types. In semiconductor detectors, response time is primarily governed by charge‐transport dynamics (Figure S7f, Supporting Information). In contrast, scintillator detectors have their response speed fundamentally limited by the radioluminescence lifetime (Figure S3g, Supporting Information). Detector architecture specifies electronic readout components depending on their type and detection mode:

1. Photon‐counting mode:
2. semiconductor detectors typically utilize charge‐sensitive preamplifiers (Figure S3h, Supporting Information);
3. scintillator‐based detectors commonly use photomultiplier tubes (Figure S3I, Supporting Information) or silicon photomultipliers;
4. Charge integration mode for both semiconductor and scintillator detectors:
5. current amplifiers (Figure S3j, Supporting Information);
6. current‐integrating amplifiers (Figure S3k, Supporting Information).

Semiconductors are connected to electronics directly, whereas scintillators require coupling through a photodetector. The rise and fall times (τ_r_, τ_f_) can be determined from the resulting output signal, observable as single‐photon counting traces. Examples include signals from a CdTe semiconductor detector^[^ ^76^ ^]^ (Figure S3l, Supporting Information) and a CsI(Tl) scintillator detector^[^ ^77^ ^]^ (Figure S3m, Supporting Information). In charge integration mode, photocurrent time traces enable evaluation of transient times (τ_tr_), encompassing or directly representing τ_r_ and τ_f_. Examples of these traces are shown as time‐of‐flight signals for an a‐Se detector^[^ ^78^ ^]^ (Figure S3n, Supporting Information) and an X‐ray modulated signal for MAPbI_3_ film^[^ ^1b^ ^]^ (Figure S3o, Supporting Information).

## 8 Spatial Analysis of Signal and Noise

**Figure 5:**
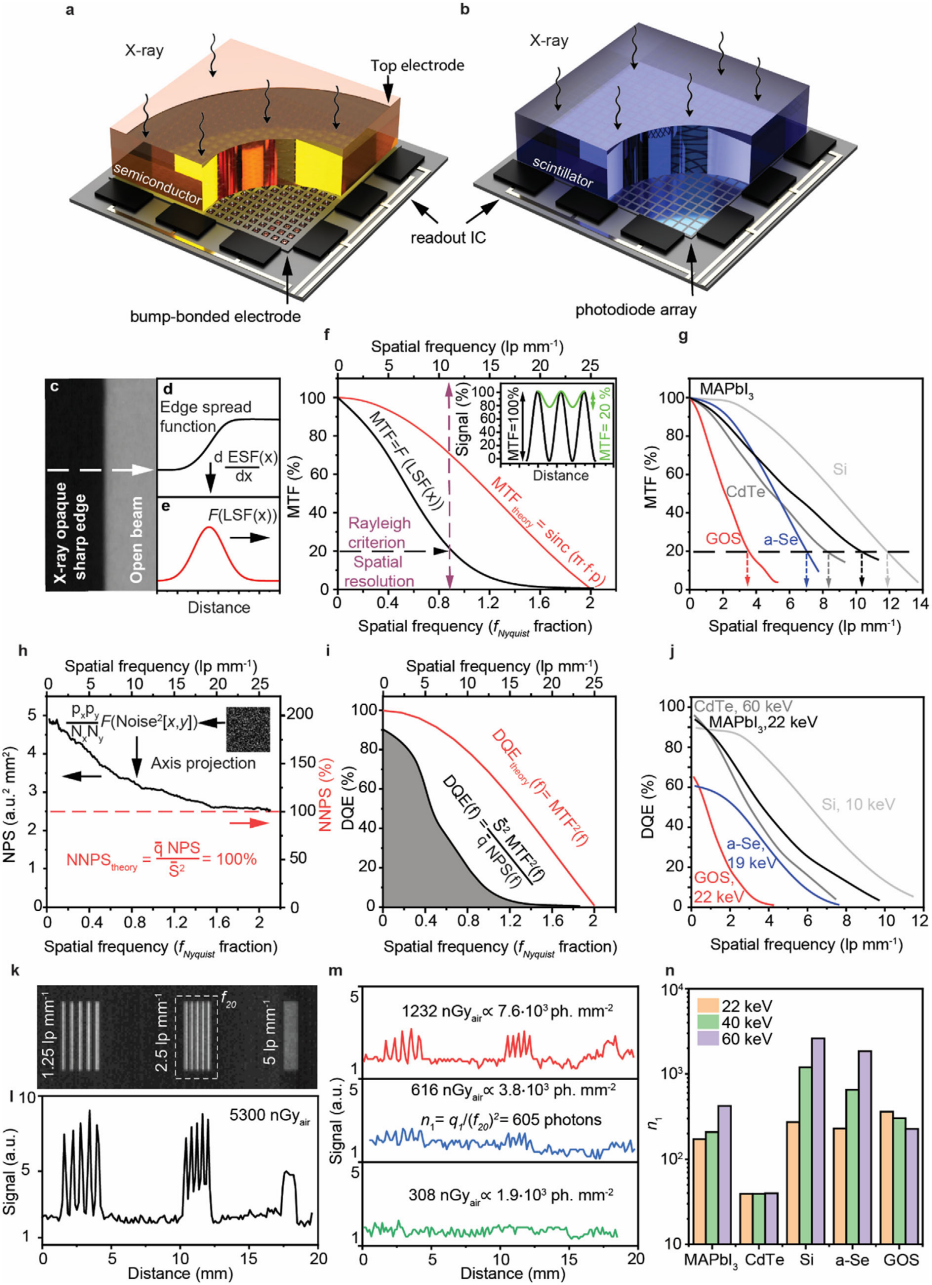
Spatial analysis of detector performance and *n*_1_ evaluation. a,b) X‐ray imaging detector structure for two major X‐ray‐to‐charge conversion technologies: direct with semiconductors, illustrated with the single crystal ball‐bonded to the readout array (a), and indirect with scintillators (b). c) X‐ray image of the slanged steel edge. d) Edge spread function vs. spatial distance, obtained from image (c) (along white arrow). d) Line spread function dependence on spatial distance, obtained as a derivative of e.f) Typical experimental (black curve) and corresponding theoretical (red curve) Modulation Transfer Function dependencies on spatial frequency. The inset shows signal modulation of a sine‐shaped object, when the Modulation Transfer Function equals 100% (black curve) and 20% (green curve). g) Dependencies of Modulation Transfer Function on spatial frequency for various detector systems based on corresponding sensor materials (CdTe—gray, MAPbI_3_—black, a‐Se—blue, GOS—red, Si—light gray). Arrows show corresponding spatial resolution values, defined as the spatial frequency at which the MTF equals 20%. h) Typical experimental Noise Power Spectrum (black solid curve, left axis) and spatial‐frequency dependence of the theoretical Normalized Noise Power Spectrum (defined by Poisson photon statistics, red dashed line, right axis). i) Typical experimental (black curve) and theoretical (red curve, defined by Poisson photon statistics and pixel pitch) Detective Quantum Efficiency dependencies on spatial frequencies. The spatial frequency in (f,h,i) is expressed in units of line‐per‐millimetre (top *X*‐axis) and normalized on the detector pitch Nyquist frequency (bottom *X*‐axis). j) Detective Quantum Efficiency dependencies on spatial frequencies calculated for various detector systems based on corresponding sensor materials ((CdTe—gray, MAPbI_3_—black, a‐Se—blue, GOS—red, Si—light gray). k) X‐ray image of a reference X‐ray opaque mask featuring slit patterns with varying spatial frequencies; the image obtained using a GOS‐based detector under 22 keV X‐ray exposure at a dose of 5.3 µGy_air_. l) Intensity profile along the horizontal axis of the slit patterns shown in (k). The limiting resolution *f*_20_ corresponds to slits with a spatial frequency of 2.5 lp mm^−1^. m) Intensity profiles of slit patterns (the same as on k,l) under reduced X‐ray doses. At a dose of 616 nGy_air_ the slit profile at the limiting spatial resolution becomes barely distinguishable. Thus, this dose corresponds to the photon fluence *q*_1_ according to Equation (22). Consequently, the figure of merit *n*_1_ is calculated according to Equation (32). n) Comparison of *n*_1_ metrics for various detector systems based on representative materials at X‐ray energies relevant to different imaging applications (22 keV for mammography, 40 keV for radiography, and 60 keV for computed tomography).

For imaging applications, detectors are typically fabricated with multiple readout channels structured as either one‐dimensional or, more commonly, two‐dimensional arrays. Semiconductor materials are connected to amplifier arrays through bump bonding (**Figure** **5**a), as seen with CdTe single crystals,^[^ ^79^ ^]^ or directly deposited onto thin‐film transistor arrays using solution‐based methods, such as MAPbI_3_ films,^[^ ^3^ ^]^ or physical vapor deposition methods, like one used for a‐Se^[^ ^80^ ^]^. Scintillator materials, by contrast, are typically coupled to photodiode arrays (Figure 5b), as exemplified by CsI(Tl) and GOS.^[^ ^81^ ^]^ The spatial analysis of signal and noise in such imaging arrays can be described by evaluating the DQE as a function of spatial frequency *f*:^[^ ^16a^ ^]^

(21) $$\mathrm{DQE}(\bar{q},\;f)=\frac{{\bar{S}}^{2}\mathrm{MT}F^{2}\left(f\right)}{\bar{q}\mathrm{NPS}\left(f\right)}$$

where MTF is a modulation transfer function, NPS is a noise power spectrum, $\bar{S}$ is the mean signal amplitude of all readout channels under homogeneous illumination by the mean photon fluence $\bar{q}$. The last can be either measured directly with a reference detector or calculated from Equation (4) as:

(22) $$\bar{q}=\frac{n_{\mathrm{photon}/\mathrm{pixel}}}{A}=\frac{D}{{\left(\frac{\mu }{\rho }\right)}_{\mathrm{air}}E_{\mathrm{ph}}}$$

which considers the known dose *D* and a monoenergetic photon beam. For a polyenergetic irradiation, the averaging over the whole X‐ray spectrum for the product ${\left(\frac{\mu }{\rho }\right)}_{\mathrm{air}}E_{\mathrm{ph}}$ is required. In Equation (21), MTF and NPS are, correspondingly, the dependencies of signal and noise amplitudes on spatial frequency.

MTF can be determined according to the slanted‐edge method.^[^ ^7^ ^]^ From the slanted‐edge profile obtained from the X‐ray transmittance image of a high‐contrast thin slice (Figure 5c), the edge spread function (ESF(x)) is derived (Figure 5d), and the MTF is then calculated as:

(23) $$\mathrm{MTF}\;\left(f\right)=F\;\left(\mathrm{LSF}\;\left(x\right)\right)=F\left(\;\frac{d}{dx}\mathrm{ESF}\left(x\right)\right)$$

where *x* is the position of a corresponding pixel. The line spread function (LSF(*x*), Figure 5e) is the derivative of the ESF(*x*); the MTF(*f*) is the spatial Fourier transform (*F*) of the LSF(*x*) (black curve in Figure 5f). From the obtained dependence MTF *vs. f*, one can estimate the spatial resolution of the detector system. By definition, MTF(*f*) corresponds to the ratio of the image modulation depth, represented by the detector system, to the original modulation depth, e.g., transmittance of the object consisting of a sine‐wave pattern with the spatial frequency *f* (inset in Figure 5d). According to the Rayleigh criterion, two neighboring points are observed as separate (i.e., optically resolved by the detection system) if the intensity signal of the image drops by at least 20% between those points.^[^ ^82^ ^]^ Thus, the spatial resolution parameter *f_20_* is defined as a spatial frequency *f* at which MTF(*f*) = 20%. To note, other intensity‐drop threshold criteria for MTF are ubiquitous, within the 10‐50% range. For the detector system with pixel pitch *p*, the theoretical MTF limit (red curve in Figure 5d) is:^[^ ^83^ ^]^

(24) $$\mathrm{MT}F_{theory}\left(f\right)=\frac{\sin\left(\pi \cdot f\cdot p\right)}{\pi \cdot f\cdot p}=\mathrm{sinc}\left(\pi \cdot f\cdot p\right)$$

Figure 5g illustrates the MTF curves obtained for various detector systems based on the corresponding sensor materials with different pixel pitches.

For a 2D array detector, NPS is calculated based on the 2D Fourier spatial transform of its noise image:^[^ ^16a^ ^]^

(25) $$\mathrm{NPS}\;\left(f_{x},f_{y}\right)=\frac{p_{x}p_{y}}{N_{x}N_{y}}F\left(\mathrm{Nois}e^{2}\left[x,y\right]\right)$$

where noise image (Noise[*x*,*y*]) can be obtained as a root‐mean‐square deviation from an average difference of *N* images under homogenous illumination with dose *D*, divided by the correction factor $\sqrt{N}$ ^[^ ^84^ ^]^; *x* and *y* are the corresponding pixel coordinates; *f_x_*, *f_y_*, *p_x_*, *p_y_, N_x_*, *N_y_* are, respectively, spatial frequencies, pixel pitch sizes, and pixel numbers along the denoted directions. When the noise is isotropic for both the *x* and *y* axes, it can be reduced to a one‐dimensional function along one detector matrix direction (black curve in Figure 5h):^[^ ^16a^ ^]^

(26) $$\mathrm{NPS}\left(f\right)=\mathrm{NP}S_{y}\;\left(f_{y}\right)=\mathrm{NP}S_{x}\;\left(f_{x}\right)=\mathrm{NPS}\;\left(f_{x},f_{y}=0\;\right)$$

We note that the accurate estimation of the NPS requires multiple averaging over different parts of the detector areas, as described in detail in ref. [85]. When the noise is determined by only the Poisson photon statistic, i.e. no contribution of the electronic noise, the normalized theoretical NPS (NNPS_theory_ = $\frac{\bar{q}\mathrm{NPS}\left(f\right)}{{\bar{S}}^{2}}$, red dashed line in Figure 5h) is equal to 1, i.e., for a single‐photon counting detector.^[^ ^86^ ^]^

Once the parameters $\bar{S}$, $\bar{q}$, MTF(*f*), NPS(*f*) are determined, the DQE vs spatial frequency can be computed (Equation 21, black curve in Figure 5i) to evaluate the overall imaging performance. According to the theoretical model (red curve in Figure 5i), for doses much higher than NED, DQE is equal to MTF^2^(*f*).^[^ ^86^ ^]^ Figure 5j illustrates the DQE curves obtained for various detector systems based on the corresponding sensor materials with different pixel pitches.

## 9 Discussion on a Unified Figure of Merit

While DQE is a crucial metric for the comprehensive assessment of the X‐ray detectors' performance, it is a complex metric that incorporates specific conditions and parameters. This complexity can make DQE somewhat challenging to comprehend and interpret, especially for researchers whose primary expertise lies outside detector characterization. In contrast, other device‐oriented research fields use simple and intuitive figures of merit (such as Power Conversion Efficiency in solar cells, Quantum Efficiency in light‐emitting diodes, and Photoluminescence Quantum Yield in quantum dots), highly instrumental in guiding the material and device optimization, comparison across reports, and technological advancement.

We therefore propose and encourage that the research community consider a new metric designed to unify the dose and spatial dependencies of DQE into a single, more intuitive figure of merit, which is easy to measure and straightforward to translate into a relevant dose for medical imaging of certain quality (i.e., contrast and resolution). Specifically, we define this metric (denoted as *n*_1_) as the minimum number of X‐ray photons passing through the smallest resolvable feature required to achieve a SNR_out_ equal to one. The procedure for determining *n*_1_​ involves two steps with array detectors (in early‐stage development, may be mimicked by scanning with a single readout channel detector). First, spatial resolution should be evaluated at a sufficiently high dose (ensuring adequate SNR) by imaging a test pattern (such as a standard line‐pair gauge or Siemens star) to identify the smallest resolvable feature (Figure 5k,l). Second, the incident dose should be gradually reduced until this smallest feature becomes indistinguishable from noise, i.e., until SNR_out_ equals one (Figure 5m). Multiplying the photon fluence corresponding to this minimal dose by the area of the smallest resolvable feature (inverse to the square of the corresponding spatial resolution) yields *n*_1_. To illustrate this approach, we apply it to a commercial GOS‐based detector (Figure 5k,l). The metrics *n*_1_ can also be expressed in DQE‐defining parameters by utilizing the DQE model to describe the dependence of *n*_photon/pixel_ on SNR_out_:

(27) $$\mathrm{DQE}\;\left(n_{\mathrm{photon}/\mathrm{pixel}},f\right)=\frac{DE}{1+\frac{\mathrm{NED}}{n_{\mathrm{photon}/\mathrm{pixel}}}}\cdot \frac{\mathrm{MT}F^{2}\left(f\right)}{NNPS\left(f\right)}$$

where NED is expressed in equivalent photon units according to Equation (8). Combining Equations (1) and (27):

(28) $$\mathrm{DQE}\;\left(n_{\mathrm{photon}/\mathrm{pixel}},f\right)=\frac{SNR_{out}^{2}}{n_{\mathrm{photon}/\mathrm{pixel}}}=\frac{DE}{1+\frac{\mathrm{NED}}{n_{\mathrm{photon}/\mathrm{pixel}}}}\cdot \frac{\mathrm{MT}F^{2}\left(f\right)}{\mathrm{NNPS}\left(f\right)}\;$$

Expressing Equation (28) rearranged into a quadratic equation for *n*_photon/pixel_:

(29) $$n_{\mathrm{photon}/\mathrm{pixel}}^{2}\frac{\mathrm{DE}\cdot \mathrm{MT}F^{2}\left(f\right)}{{\mathrm{SNR}}_{\mathrm{out}}^{2}\cdot \mathrm{NNPS}\left(f\right)}-n_{\mathrm{photon}/\mathrm{pixel}}-\mathrm{NED}=0$$

Solving Equation (29) for *n*_photon/pixel_ and keeping only the physically meaningful (positive) root gives the dependence of *n*_photon/pixel_ on SNR_out_:

(30) $$n_{photon/pixel}\left(SNR_{out}\right)=\frac{1+\sqrt{1+\frac{4\mathrm{DE}\cdot \mathrm{MT}F^{2}\left(f\right)\cdot \mathrm{NED}}{{\mathrm{SNR}}_{\mathrm{out}}^{2}\cdot \mathrm{NNPS}\left(f\right)}}}{2\frac{\mathrm{DE}\cdot \mathrm{MT}F^{2}\left(f\right)}{{\mathrm{SNR}}_{\mathrm{out}}^{2}\cdot \mathrm{NNPS}\left(f\right)}}$$

According to the *n*_1_ definition, the corresponding *n*_photon/pixel_ is evaluated at SNR_out_ = 1. Assuming that the smallest resolvable feature size corresponds to *f_20_* and, for simplicity, NNPS(*f*) is frequency independent and equal to 1, Equation (30) simplifies to:

(31) $$\begin{array}{ccc} n_{\mathrm{photon}/\mathrm{pixel}}\left(\mathrm{SN}R_{\mathrm{out}}=1\right) & = & \frac{1+\sqrt{1+4\cdot {\left(0.2\right)}^{2}\cdot \mathrm{DE}\cdot \mathrm{NED}}}{2\cdot \mathrm{DE}\cdot {\left(0.2\right)}^{2}}\\ & = & \frac{1+\sqrt{1+0.16\cdot \mathrm{DE}\cdot \mathrm{NED}}}{0.08\cdot \mathrm{DE}} \end{array}$$

Then, according to the *n*_1_ definition, it is equal to the corresponding photon fluence *q_1_* (*n*_photon/pixel_ divided by pixel area, *A*) multiplied by the smallest resolvable feature size, which is inversely proportional to (*f_20_)* ^2^. This yields final expressions:

(32) $$n_{1}=\frac{q_{1}}{f_{20}^{2}}=\frac{n_{\mathrm{photon}/\mathrm{pixel}}\left(\mathrm{SN}R_{\mathrm{out}}=1\right)}{A\cdot f_{20}^{2}}$$

(33) $$n_{1}=\frac{1+\sqrt{1+0.16\cdot \mathrm{DE}\cdot \mathrm{NED}}}{0.08\cdot \mathrm{DE}\cdot A\cdot f_{20}^{2}}$$

We compared *n*_1_ across several detector examples, including commercial imagers and research prototypes, covering energy ranges relevant to mammography, general radiography, and computed tomography in Figure 5n. We propose that researchers studying novel X‐ray detector materials and interested in their benchmarking will report *n*_1_, whether it is determined experimentally using the two‐step approach described previously or through the DQE model (Equation 32). We hope that the X‐ray detector community adopts *n*_1_ as a standard figure of merit. By opting for reporting *n*_1_, the research community can foster more direct and fair comparisons of low‐dose imaging performance, encouraging competitive advancements similar to those that have accelerated progress in other research areas, such as solar cells, light‐emitting diodes, and quantum dots. Noteworthy, *n*_1_​ can also be applied to early‐stage detectors that may not yet exist in full‐array configurations, enabling researchers to assess feasibility before investing in costly fabrication processes.

## 10 Conclusion

We anticipate that the presented DQE‐based characterization framework will facilitate the objective evaluation and comparison of novel X‐ray detector materials, especially when the research is motivated by potential applications in medical imaging. For X‐ray detector performance assessment, it is important to establish proper characterization conditions (including X‐ray source collimation, energy spectrum, readout electronics, etc.) commensurate with the intended application area (e.g., computed tomography, mammography, or radiography). Even though specific applications may require different conditions, the described characterization methods can be applied universally for the performance comparison of diverse detectors within a designated X‐ray medical imaging area. We emphasize a new convenient figure of merit *n*_1_, which is intuitive and easily assessable from the experiment parameter that integrates both radiation dose and spatial resolution aspects of DQE. Unlike sensitivity or the lowest detectable dose, which can be arbitrarily improved by adjusting electric field strength or integration time, *n*_1_ is highly relevant for low‐dose imaging and DQE‐consistent, straightforwardly evaluated from images of the standard slit pattern at the limiting spatial frequency (f_20_). This directly reflects the dose–resolution trade‐off that is central for patient imaging (a lower *n*_1_ corresponds to a lower dose for the same resolvable feature size). Moreover, *n*_1_ is reproducible and comparable across detector technologies, as it is derived from standard X‐ray imaging of slit or star‐pattern phantoms and remains valid for both direct and indirect detectors in charge‐integration or photon‐counting modes. For the evaluation routine, we provide a ready‐to‐use calculator for these characteristics in several formats: as a Mathcad worksheet (Supporting Information), a MATLAB application, and as an interactive website.^[^ ^20^ ^]^ Table S1 (Supporting Information) briefly summarizes all characteristics recommended for reporting, while Table 2 provides a detailed characterization checklist. To assist in benchmarking, estimated figures of merit are provided for examples of CdTe, GOS, a‐Se, and MAPbI_3_ detectors in Table S2 (Supporting Information). Technical parameters and corresponding measurement conditions for these detectors are summarised in Table S3 (Supporting Information).

## Conflict of Interest

The authors declare no conflict of interest.

## Author Contributions

K.S., V.B., G.J.M., Y.Z., and J.Z. researched data for the article. K.S. analyzed the detective quantum efficiency metrics and formulated the n_1_ concept. Y.Z., J.Z., and J.H. contributed to temporal response characterization methods. K.S. and V.B. created calculation tools. K.S., S.Y., and M.V.K. wrote the manuscript with contributions from all authors.

Mathcad worksheet tutorial: https://youtu.be/b1Fb‐BmKCtY

MATLAB software tutorial: https://youtu.be/RGa9SugcS18

Website tutorial: https://youtu.be/r9I82cdtKuU

MATLAB application codes are available in a public GitHub repository: https://github.com/VitaliiBartoshETH/perovXimager.git.

## Supporting information



Supporting Information

Supporting Information

## Data Availability

Step‐by‐step calculations of the protocol described in this Perspective on examples of typical X‐ray detector materials (CdTe, Si, a‐Se, GOS, MAPbI_3_) are available as Mathcad worksheets in Supplementary Data, MATLAB application in a public GitHub repository, and a non‐proprietary online version of the calculation tool at the link in ref. [20]. The tutorial videos, describing data processing and calculation processes, are available at the KovalenkoLab YouTube channel under the following links:
