# Supplementary material for: Characterizing Emerging Detector Materials for Low‐Dose X‐Ray Imaging
Source: Adv Mater. 2025 Sep 11;37(45):e12795. doi: 10.1002/adma.202512795 (PMC12483158; doi:10.1002/adma.202512795)
Supplement: Supplementary file 1 — Supporting Information [file ADMA-37-e12795-s001.pdf]

ADVANCED MATERIALS

Supporting Information

for *Adv. Mater.*, DOI 10.1002/adma.202512795

Characterizing Emerging Detector Materials for Low-Dose X-Ray Imaging

Kostiantyn Sakhatskyi, Vitalii Bartosh, Ying Zhou, Gebhard J. Matt, Jingjing Zhao, Sergii Yakunin, Jinsong Huang* and Maksym V. Kovalenko**

Supporting Information

Characterizing emerging detector materials for low-dose X-ray imaging

Kostiantyn Sakhatskyi[1,2], Vitalii Bartosh[1,2], Ying Zhou[3], Gebhard J. Matt[1,2], Jingjing Zhao[4], Sergii Yakunin[*1,2], Jinsong Huang[*3,5], Maksym V. Kovalenko[*1,2,6]

[1] Laboratory of Inorganic Chemistry, Department of Chemistry and Applied Biosciences, ETH Zürich, CH-8093 Zürich, Switzerland

[2] Laboratory for Thin Films and Photovoltaics, Empa – Swiss Federal Laboratories for Materials Science and Technology, CH-8600 Dübendorf, Switzerland

[3] Department of Applied Physical Sciences, University of North Carolina at Chapel Hill, Chapel Hill, NC, USA

[4] School of Physical Science and Technology, Chongqing Key Lab of Micro&Nano Structure Optoelectronics, Southwest University, Chongqing, 400715 China.

[5] Department of Chemistry, University of North Carolina at Chapel Hill, Chapel Hill, NC, USA

[6] Institute of Energy Science and Technology (SIEST), Sungkyunkwan University (SKKU)
2066, Seobu-ro, Jangan-gu, Suwon, Gyeonggi-do 16419, Republic of Korea

[*]E-mail: mvkovalenko@ethz.ch; jhuang@unc.edu; yakunins@ethz.ch

Contents

Fig. S1. Characterising signal and noise dependencies on radiation dose. 3

Fig. S2. Performance metrics as a function of X-ray energy. 4

Fig. S3. Characterisation methods of temporal response. 5

Table S1. X-ray detectors' figures of merit. 6

Table S2. Summary of estimated X-ray detectors' figures of merit with example detector materials. 6

Table S3. Summary parameters and measurement conditions with example detectors. 6

Supporting Information References 7

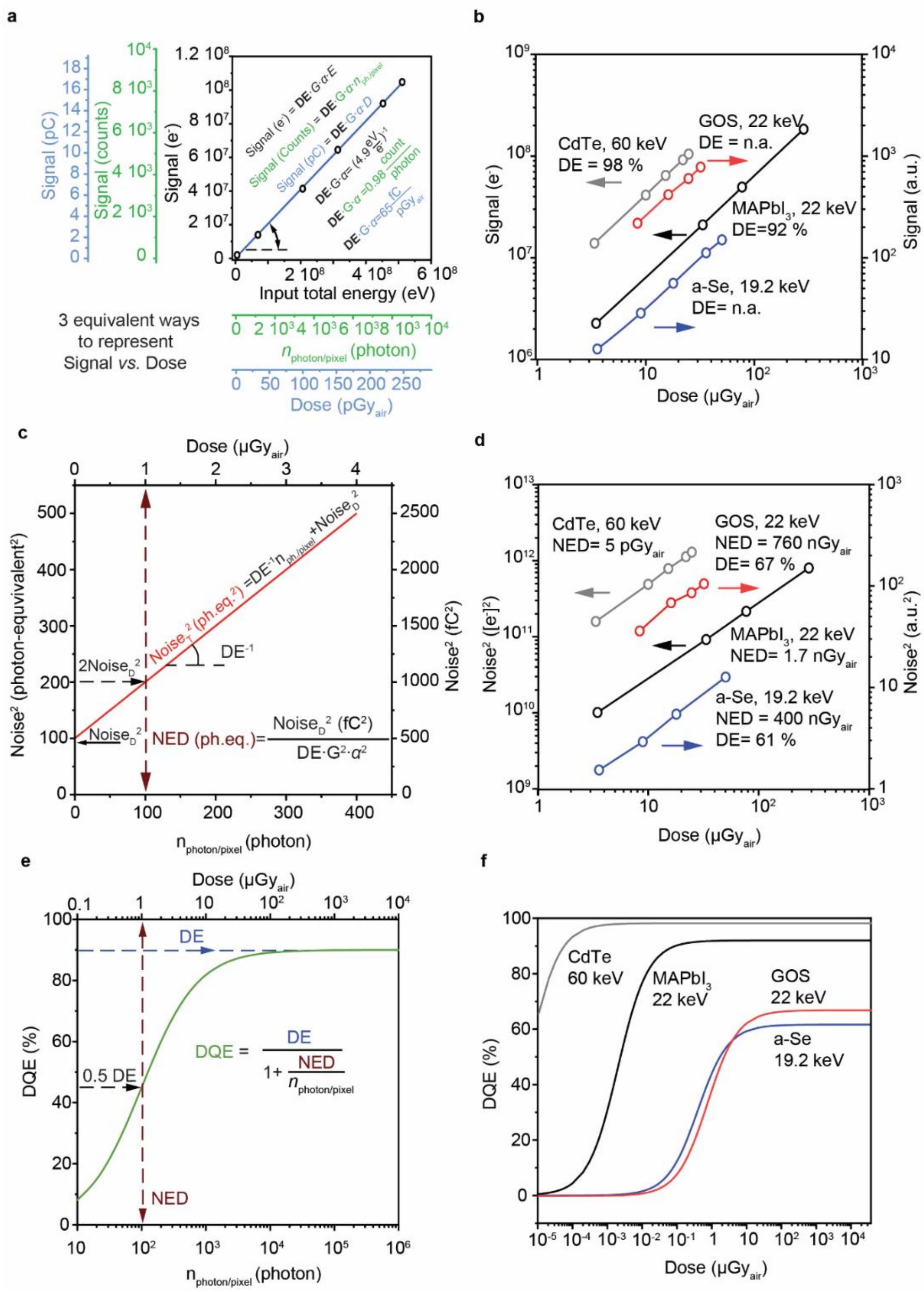

Fig. S1. Characterizing signal and noise dependencies on radiation dose. a, Signal amplitude dependence on the radiation dose expressed in 3 equivalent representations in terms of signal and dose units. The signal is expressed in charge units (Coulomb, C and electrons, e^-) and counts. Dose units are expressed as total input energy in eV, number of X-ray photons and absorbed dose in Gray in air. **b,** Examples of dependencies of signal amplitude on radiation dose for various detector materials (CdTe – grey, methylammonium lead triiodide ($MAPbI_3$) – black, a-Se – blue, gadolinium oxysulfide (GOS) – red) at different X-ray energies. Detection efficiency (DE) values are indicated where they can be directly determined from these relationships; for datasets where direct determination is not possible, DE is marked as "n.a.". **c,** Dependence of the squared total noise, expressed in photon-equivalent (left Y-axis) and in arbitrary units (right Y-axis), on radiation dose. **d,** Examples of dependencies of the squared total noise, expressed in electrons (left Y-axis) and in arbitrary units (right Y-axis), on radiation dose for various detector materials (CdTe – grey, $MAPbI_3$ – black, a-Se – blue, GOS – red) at different X-ray energies. NED and DE values are indicated where they can be directly determined from these relationships. **e,** Dependence of the Detective Quantum Efficiency on radiation dose, based on a model for a single-readout-channel detector, shown as the inset equation. The radiation dose in **c,e** is expressed in Gray in air units (top X-axis) and X-ray photon number (bottom X-axis). **f,** Examples of Detective Quantum Efficiency dependencies on dose for various detector materials (CdTe – grey, $MAPbI_3$ – black, a-Se – blue, GOS – red) at different X-ray energies.

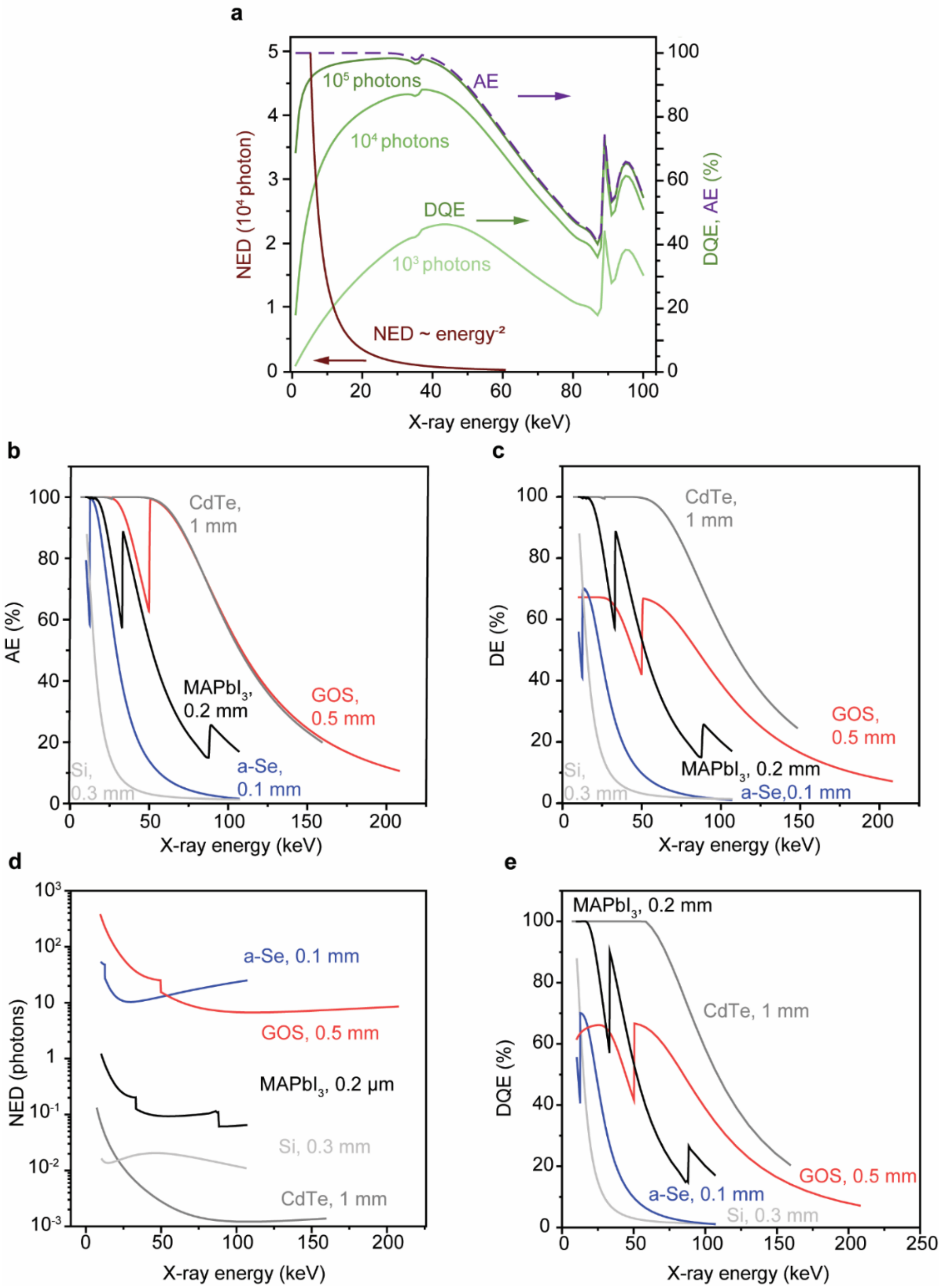

Fig. S2. Performance metrics as a function of X-ray energy. a, Typical dependencies of the Noise Equivalent Dose (brown curve, left Y-axis) and Detective Quantum Efficiencies, Absorption Efficiency (green curves and dashed violet curve correspondingly, right Y-axis) on X-ray energy. Detective Quantum Efficiency is shown for different photon numbers (doses). **b-e,** Dependencies of the Absorption Efficiency (**b**), Detection Efficiency (**c**), Noise Equivalent Dose (**d**) and Detective Quantum Efficiency (**e**) on X-ray energy for various detector materials (CdTe – grey, $MAPbI_3$ – black, Si – light grey, a-Se – blue, GOS – red) with indicated thicknesses.

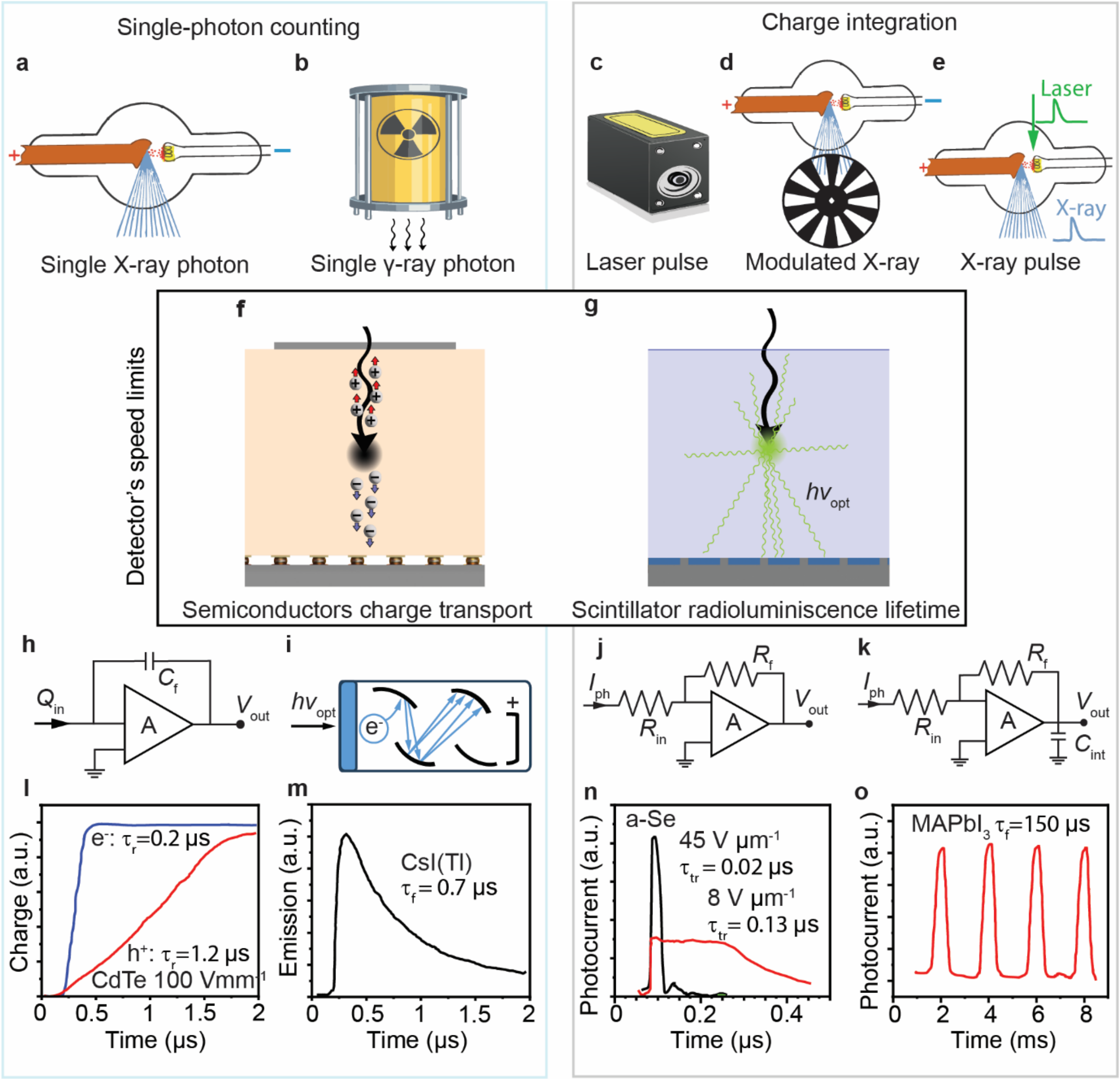

Fig. S3. Characterisation methods of temporal response. **a-e,** Excitation sources for detector speed evaluation: X-ray tube at low flux (**a**), gamma-ray emitting radioactive isotopes (**b**), pulsed laser (**c**), X-ray tube modulated by X-ray opaque chopper wheel (**d**), laser-triggered pulsed X-ray tube (**e**). **f, g** Schematics of processes limiting speed in semiconductor direct conversion detectors (**f**) and scintillators (**g**). **h-k,** Readout electronics schematics utilised for various types of detectors: charge-sensitive preamplifier (**h**), photomultiplier tube (**i**), current amplifier (**j**) and current-integrating amplifier (**k**). **l-o**, Examples of signal traces, utilised for temporal response characterisation. Photon-counting traces for CdTe detector[1] (**l**) for electron (blue) and hole (red) collection. Single-photon detection trace with CsI(Tl) scintillator[2] (**m**). Time-of-flight traces for a-Se[3] (**n**). Modulated X-ray photocurrent trace obtained with $MAPbI_3$ film[4] (**o**).

Table S1. X-ray detectors' figures of merit.

Figure of merit	Estimated from	What is characterised
**Noise equivalent dose**	Noise *vs.* dose	Low-dose performance
**Detection efficiency**	Signal *vs.* dose, noise *vs.* dose	Fraction of detected photons
**X-ray energy at 50% absorption efficiency**	Absorption efficiency *vs.* X-ray energy	Applicable X-ray energy range
**Rise and fall times or cut-off frequency**	Signal *vs.* time	Detector speed
**Spatial resolution**	Modulation transfer function *vs.* spatial frequency	The smallest resolvable spatial feature
**Number of photons passing through the smallest resolvable feature to achieve SNR=1**	Images of limiting spatial resolution feature *vs.* different dose, or calculated from spatial resolution, detection efficiency and noise equivalent dose.	The detector's low-dose imaging performance

Table S2. Summary of estimated X-ray detectors' figures of merit with example detector materials.

Figure of merit	GOS	a-Se	$MAPbI_3$	Si	CdTe
Noise equivalent dose (Gy_{air})	$7.6\cdot10^{-7}$	$4\cdot10^{-7}$	$1.74\cdot10^{-9}$	$4\cdot10^{-9}$	$5\cdot10^{-12}$
Noise equivalent dose (photon-equivalent)	79	12	0.4	0.017	0.002
Detection efficiency (%)	67	61	92	90	98
X-ray energy at 50% absorption efficiency (keV)	110	30	52	15	110
Rise and fall times or cut-off frequency (s)	0.7	0.2	$2\cdot10^{-5}$	$7\cdot10^{-8}$	$8.7\cdot10^{-8}$
Spatial resolution (lp mm^{-1})	3.5	7	10	12	8
n_1 (photons)	368	210	172	64	40

Table S3. Summary parameters and measurement conditions with example detectors.

Parameter	GOS	a-Se	$MAPbI_3$	Si	CdTe
Average X-ray energy (keV)	22	19.2	22	10	60
Detector thickness (mm)	0.5	0.1	0.2	0.3	1
Pixel area (μm^2)	17689	4900	1600	3025	10000
Pixel pitch (µm)	133	70	40	55	100
Reference detector	RM1426 Teledyne (pixels binned 7 times)	Ref[5] and Ref[6] (MTF)	Ref[7]	Advacam MiniPIX SPRINTER	Kromek, Dectris PILATUS3 X (MTF)

Supporting Information References

1. Nakhostin, M. Optimizing timing performance of CdTe detectors for PET. *Phys Med Biol* **62**, N485-n505 (2017).
2. Fast light of CsI(Na) crystals. *Chinese Physics C* **35**, 1130 (2011).
3. Abbaszadeh, S., Scott, C. C., Bubon, O., Reznik, A. & Karim, K. S. Enhanced Detection Efficiency of Direct Conversion X-ray Detector Using Polyimide as Hole-Blocking Layer. *Scientific Reports* **3**, 3360 (2013).
4. Yakunin, S. *et al.* Detection of X-ray photons by solution-processed lead halide perovskites. *Nat. Photonics* **9**, 444-449 (2015).
5. Zhao, B. & Zhao, W. Imaging performance of an amorphous selenium digital mammography detector in a breast tomosynthesis system. *Med Phys* **35**, 1978-1987 (2008).
6. Kim, Y. C. *et al.* Printable organometallic perovskite enables large-area, low-dose X-ray imaging. *Nature* **550**, 87-91 (2017).
7. Sakhatskyi, K. *et al.* Stable perovskite single-crystal X-ray imaging detectors with single-photon sensitivity. *Nat. Photonics* **17**, 510-517 (2023).
